# An integrated surgical protocol for adult patients with hemifacial microsomia: Methods and outcome

**DOI:** 10.1371/journal.pone.0177223

**Published:** 2017-08-04

**Authors:** Kazuaki Yamaguchi, Daniel Lonic, Ellen Wen-Ching Ko, Lun-Jou Lo

**Affiliations:** 1 Plastic & Reconstructive Surgery, and Craniofacial Research Center, Chang Gung Memorial Hospital, Chang Gung University, Taoyuan, Taiwan; 2 Department of Craniofacial Orthodontics, and Craniofacial Research Center, Chang Gung Memorial Hospital, Chang Gung University, Taoyuan, Taiwan; Medical University of South Carolina, UNITED STATES

## Abstract

**Background:**

Hemifacial microsomia (HFM) features hypoplasia and asymmetry in skeletal as well as soft tissue, and correction of the deformity is difficult in terms of aesthetic outcome. The purpose of this study is to examine the validity of an integrated treatment protocol for correction of this facial deformity.

**Patients and methods:**

A retrospective study was performed on adult HFM patients who received two-jaw orthognathic surgery combined with facial contouring procedures in the first stage, and fat injection for the residual facial deficiency in the second stage. Inclusion criteria were patients treated by the same surgeon and follow-up at least 6 months. The demographic, perioperative, and follow-up data were collected. We defined a facial surface area discrepancy index (FDI) for objective assessment of the symmetry between the affected and non-affected side, and utilized visual analogue scale (VAS) for subjective evaluation of facial asymmetry before and after surgical treatment.

**Results:**

A total of 14 patients were included. The mean age at orthognathic surgery was 21.7 years. Four patients were categorized as Pruzansky-Kaban type I, while the remaining 10 patients were type II (7 patients type IIA, 3 patients type IIB). Fat injection as a secondary procedure was performed in eleven cases (79%). The mean pre- and postoperative FDI was 87.6±6.3 and 95.4±5.2 with a significant advance for symmetry (*p <* 0.001). The pre- and postoperative VAS for asymmetry was 7.2±1.7 and 3.8±2.4 respectively, with a significant improvement (*p* = 0.002).

**Conclusion:**

Our integrated approach using orthognathic surgery, facial contouring surgery and subsequent fat injection is satisfactory and obtain significant improvement of the facial deformity considering the complexity of HFM.

## Introduction

Hemifacial microsomia (HFM) is characterized by an asymmetric face that results from developmental impairment of the first and second branchial arches, affecting the cheek, chin, mouth, ear, and/or eye. It is one of the common craniofacial malformations occurring in approximately 1 in 5500 live births [1]. In genetic aspect, most cases are sporadic occurrence, but some have familial tendency exhibiting autosomal dominant trait [2, 3]. Goldenhar syndrome is part of this spectrum, which can involve internal organs and vertebrae [4]. The possible etiology, although unknown, is thought to be a vascular disruption during embryonic development at about one month of gestation with sporadic occurrence. The hypovascularity is believed to cause hypoplasia or absence of facial muscles with facial nerve involvement as well as absence of the parotid gland and masticatory muscles. The orbital and occlusal level can be canted due to hypoplasia of the maxillary and mandibular arches on the affected side. Ear deformity may also be present as microtia, accessory preauricular tags, and/or middle ear defects with hearing impairment. A recent meta-analysis revealed no difference in the incidence of HFM in gender and right/left frequency [5]. Team care is essential for the management of patients with HFM [6]. A previous cross-sectional study suggested that HFM was associated with an elevated risk for psychosocial difficulties in childhood, similar to other craniofacial conditions [7]. This can translate into significant psychological burden and social problems affecting the patient’s quality of life [8].

Rib grafts, distraction osteogenesis of the hypoplastic mandible, and microtia surgery have been reported as surgical treatments for the facial deformity of HFM [9, 10]. However, these treatment methods do not completely solve the esthetic problem. Reports about two-jaw orthognathic surgery (OGS) for HFM have been limited in the literature. Considering the quality of life of HFM patients, it is crucial to address the facial deformity in order to achieve an aesthetically satisfying outcome. We have developed a two-stage treatment protocol for adult patients with HFM including bone surgery and soft tissue management. The first step is to correct the osseous asymmetry using two-jaw OGS combined with facial contouring procedures. The remaining facial deficiency is augmented using a microautologous fat transplantation technique in the second stage [11]. This study evaluated the outcome after the treatment protocol with regard to the facial appearance and symmetry in HFM patients.

## Patients and methods

This retrospective study received approval from the Institutional Review Board, Chang Gung Medical Foundation (IRB number: 102-5354B). Written informed consents were obtained from the patients or parents. The 2-staged treatment protocol was applied to all patients with HFM who had finished their growth spurt and had functioning temporomandibular articulation. A chart review included all adult HFM patients who underwent OGS and facial contouring procedures. The patients were subdivided according to the Pruzansky-Kaban classification [12, 13]. The surgeries were conducted by a single surgeon (L.J.L.) at the Craniofacial Center, Chang Gung Memorial Hospital between July 1, 2003 and December 31, 2014. We excluded the following patients: 1) Pruzansky-Kaban classification type III, 2) follow-up less than 6 months after the last surgery, 3) incomplete or missing data, and 4) patient rejection to be included in the study. Using these criteria, 14 HFM patients were included in the analysis and categorized by age at the surgery, side of microsomia, gender, previous surgery, Pruzansky-Kaban classification, type of surgery, pre-/postoperative facial surface area discrepancy index (FDI), and visual analogue scale (VAS) for pre-/postoperative facial asymmetry.

### Surgical planning

**Fig 1:**
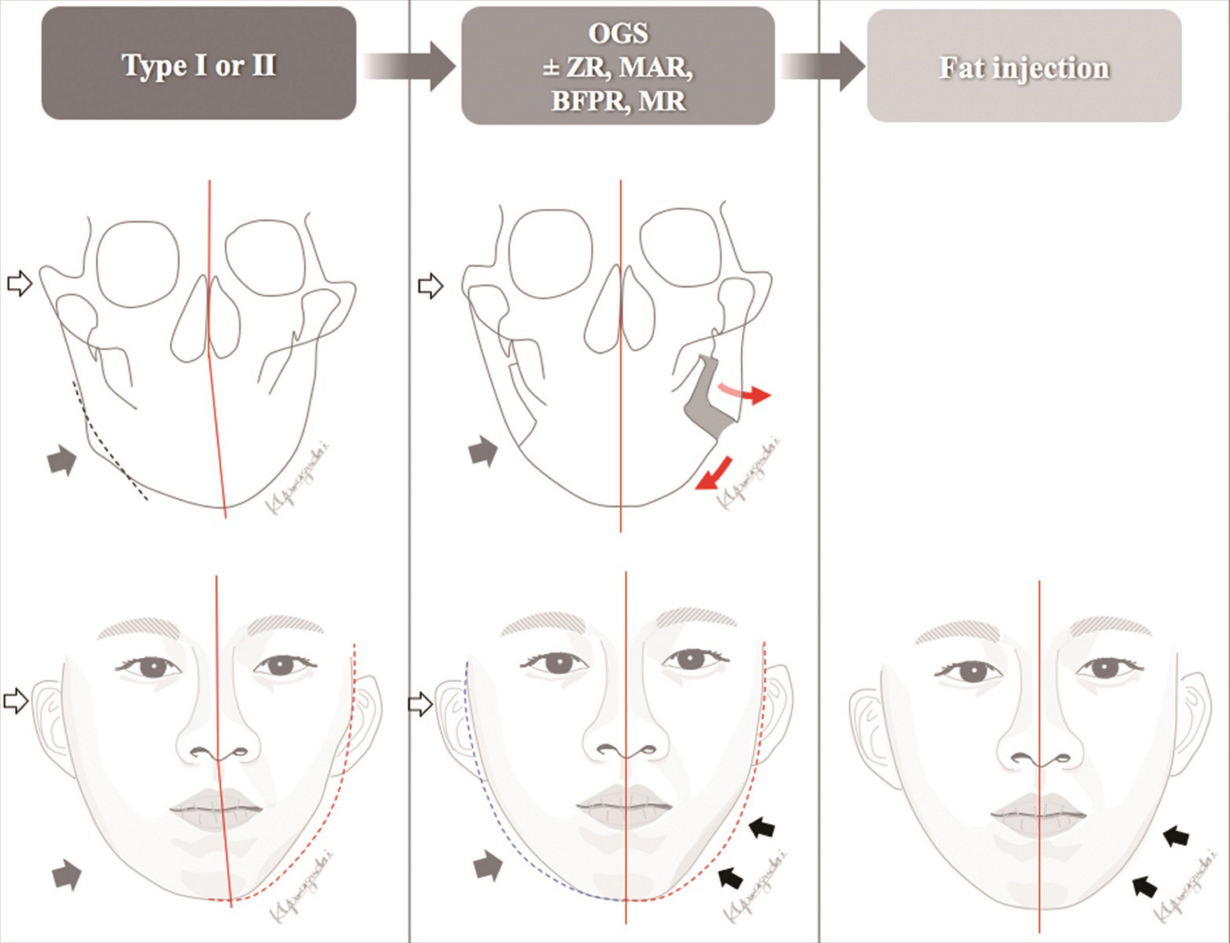
Treatment scheme. (Left, upper and lower) Preoperative frontal cephalogram and face. White and gray arrows mark protruded zygoma and mandibular angle, respectively. The lower facial midline is deviated to the affected side. The dotted red line shows the mirror image of the jaw line. (Middle, upper and lower) Postoperative frontal cephalogram and face, status after 1^st^ stage of correction. White and gray arrows show reduction of zygoma and mandibular angle, respectively. The bone gap between the mandibular segments is maintained to augment the jaw line of the left affected side. Red arrows indicate the movement of each segment. The asymmetric deformity along the affected jawline can be accentuated after OGS (black arrows). (Right) The final result after the 2^nd^ stage of treatment. Fat injection could address the deficient area after OGS to obtain symmetry. **Abbreviations**: BFPR, buccal fat pad removal; MAR, mandibular angle resection; MR, masseter muscle reduction; Type, Pruzansky-Kaban classification; OGS, orthognathic surgery; ZR, zygoma reduction.

The OGS planning was started by the orthodontists using conventional methods of patient photos, cephalometric tracing, dental stone models, and face bow transfer. The OGS planning routinely included LeFort I osteotomy, bilateral sagittal split osteotomy, and genioplasty in this study of HFM patients. Computed tomography (CT) or cone-beam CT were later used for further evaluation, modification of planning, and three-dimensional (3D) simulation. The Pruzansky-Kaban classification was reevaluated in 3D CT because of higher accuracy than the combination of cephalogram and panorex x-rays [14]. 3D simulation provides the precise position for the maxillary and mandibular segments [15]. The patient’s image is oriented in a balanced position regarding the eyes, orbits, nasal dorsum, and natural head position. Appropriate yaw and roll rotation of the 3D image of maxillomandibular complex provides a symmetric configuration of the facial skeleton and helps to avoid premature contact and bony collisions between the proximal and distal mandibular segments. To achieve cheek symmetry, the proximal segments of the mandibular ramus are adjusted in a vector of in-/outward direction to balance the soft tissue discrepancy with respect to the relationship between the condyle and the glenoid fossa. In a usual way, the affected and less protruded mandibular ramus can be augmented through maintaining the bone gap between the proximal and distal segments (Fig 1). On the other hand, the bone gap or the collision at the non-affected side should be reduced to decrease the fullness in the ramus area. Bone contouring and reduction of the contralateral zygoma and mandibular angle as well as sliding genioplasty are outlined in the 3D plan in order to reduce tissue discrepancy and increase the symmetry between the affected and the contralateral side [16].

### Surgical technique- orthognathic surgery

In our center, the single-splint method for two-jaw surgery is the standard procedure. A bilateral sagittal split osteotomy for the mandible and a LeFort I osteotomy for the maxilla were performed in the standard fashion. The two jaw segments were mobilized, and the intermaxillary fixation was performed using the final occlusal splint. The maxillomandibular complex was moved by translation and rotation in three dimensions to the new intended position as a single unit. It was often that the maxilla was shortened on the contralateral side and lengthened on the microsomia side in order to level the occlusal plane. Yaw rotation of the maxillomandibular complex was determined by the 3D simulation. Intraoperative evaluation was performed to check the dental midline, occlusal plane, teeth show from upper lip, and overall facial symmetry. The bony segments were then fixed with four titanium plates for the maxilla, and three transbuccal bicortical screws on each side for the mandible. In maintaining the bony gap between the proximal and distal segments, the space was kept by utilizing a small bone chip during the ramus bicortical fixation. After completion of the bone fixation, the bone chip was removed. As indicated, simultaneous mandibular angle reduction in the contralateral side was accomplished under direct vision prior to ramus fixation. The genioplasty was performed to balance the aesthetic E-line and midline [17]. Chin horizontal osteotomy was done below the mental foramen from a lower vestibule incision. After confirmation of advancement and midline position, rigid fixation was performed with two titanium plates. Adjunct procedures such as zygomatic body and arch reduction is performed in combination with buccal fat removal and masseter muscle reduction to control the soft tissue discrepancy in the first stage [18].

### Surgical technique- soft tissue augmentation

Soft tissue augmentation was recommended for all patients who had a soft tissue deficiency at least six months after the OGS. In the second stage, it is important to assess the soft tissue distribution in the sitting position considering the gravity effect before the surgery. Under general anesthesia, fat is aspirated from the lower abdomen using a 16-gauge Coleman cannula [19]. The microautologous fat transplantation technique is then applied for injection to balance the facial symmetry [11].

### Facial surface area discrepancy index (FDI)

**Fig 2:**
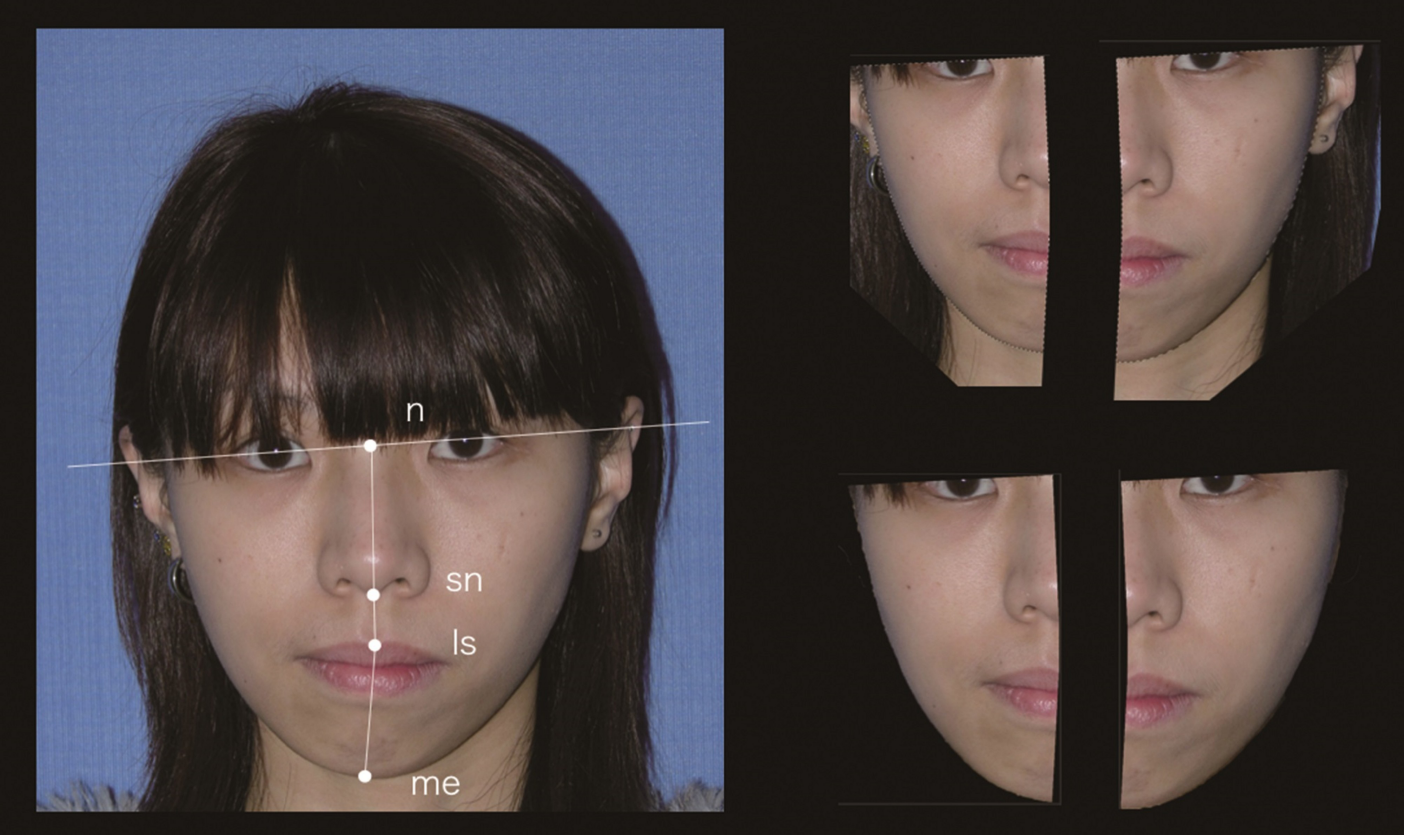
Evaluation of facial surface area discrepancy index (FDI). FDI = affected side area / non-affected side area. Digitized facial landmarks: n, nasion; sn, subnasale; ls, labiale superius; me, menton.

Objective facial symmetry assessment was developed and carried out on the standardized frontal view photo. Digitized facial midline landmarks including nasion (n), subnasale (sn), labiale superius (ls), and menton (me) were marked. The line connecting the facial landmarks (n-sn-ls-me) was defined as a central reference line (Fig 2). The mid-lower facial area was outlined as the part surrounded by the bipupillary line, the central reference line, and the cheek margin. Using Photoshop CS 6.0 (Adobe Systems, San Jose, CA), the surface area was calculated on both sides. FDI was defined as the ratio between the areas on the affected side over the non-affected side to evaluate the area difference ratio. The measurement of the FDI for pre-/postoperative appearance was repeated three times with a day interval between each measurement. The intra-rater reliability was assessed using intraclass correlation coefficient (ICC).

### Evaluation of asymmetry

**Fig 3:**
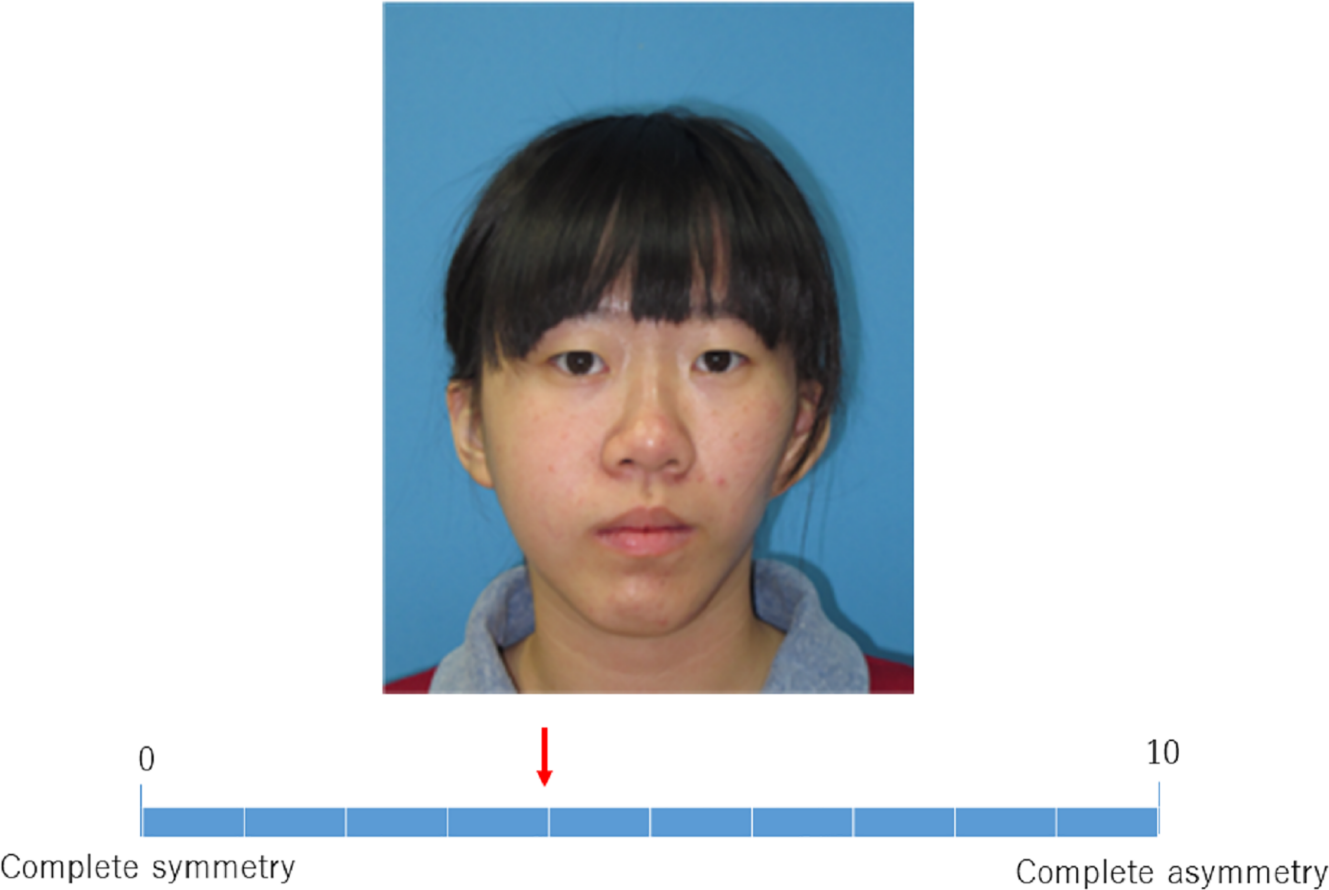
Performing the visual analogue scale (VAS) on one of the patient photographs. The scale is from 0 (complete symmetry) to 10 (complete asymmetry). The rater move the red arrow on the gridline.

To perform subjective assessment for facial asymmetry, three board-certified plastic surgeons and three research assistants scored the visual analogue scale (VAS) for pre-/postoperative clinical pictures. The VAS for asymmetry consisted of a 10cm line with zero on one end, representing complete symmetry, and 10 on the other end representing complete asymmetry (Fig 3 and S1 File). The inter-observer reliability was assessed using Cronbach’s alpha reliability coefficient.

### Statistical analysis

Descriptive statistics were presented as the mean and standard deviation (SD) for continuous variables. Paired t-test was used to compare the pre-/postoperative FDI. Pre-/postoperative VAS were assessed using the Mann-Whitney *U* test. P value less than 0.05 was considered to be significantly different. All statistical analyses were done using SPSS statistic software (Version 21.0; IBM, Armonk, NY).

## Results

**Fig 4:**
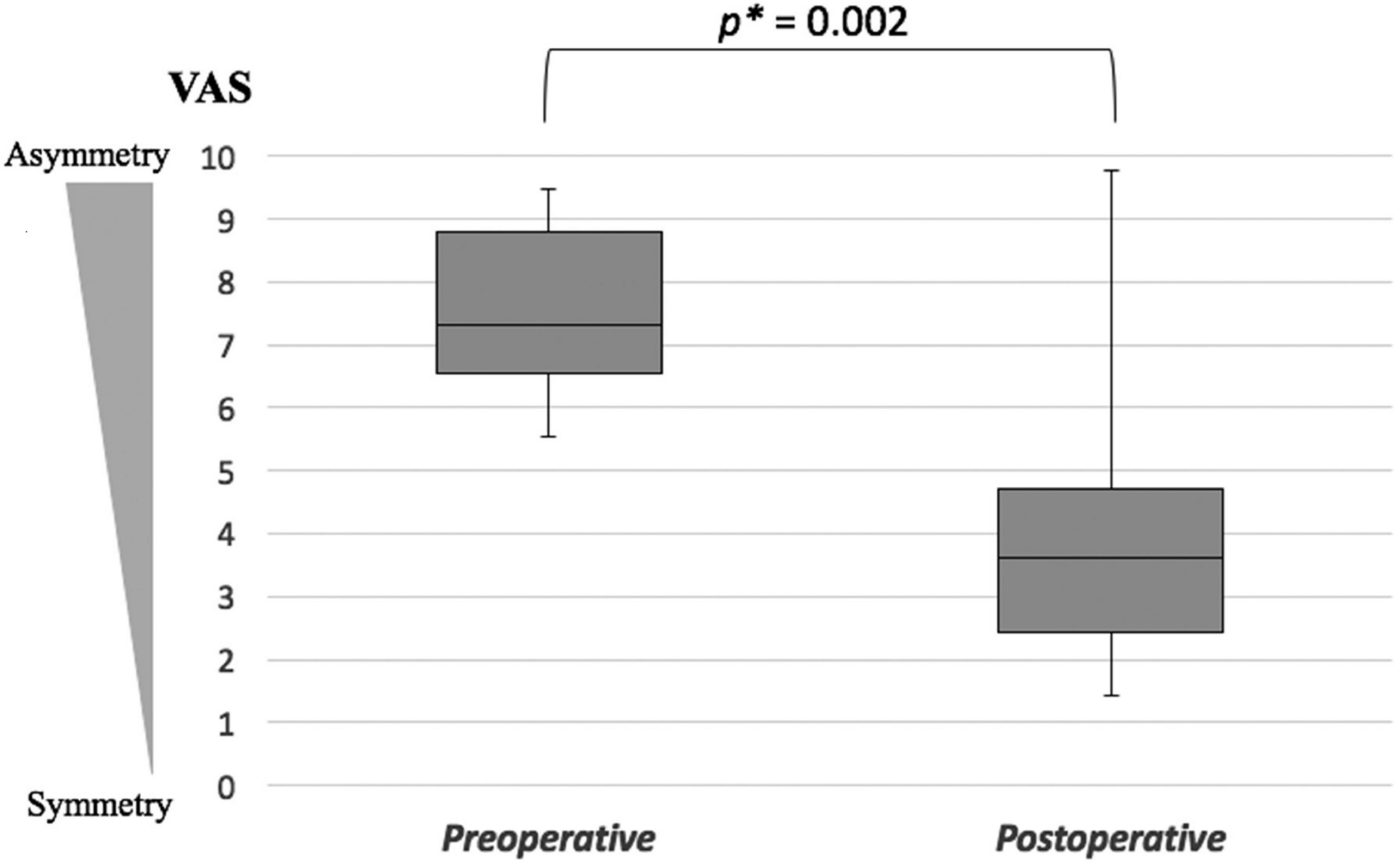
Pre- and postoperative visual analogue scale (VAS). *: Mann-Whitney *U* test.

The patient demography including age at the time of surgery, laterality, gender, Pruzansky-Kaban classification, type of surgery, and pre-/postoperative FDI is described in Table 1. The mean age at OGS surgery was 21.7 years (SD 3.8). The mean follow-up duration after OGS and fat injection was 25.8 months (SD 11.8) and 16.8 months (SD 10.3) respectively. Four patients were categorized as type I, while the remaining 10 patients were type II (7 patients type IIA, 3 patients type IIB). Two patients (case 5, 13) had distraction osteogenesis during the growing age, but had residual facial asymmetry after the growth spurt. Fat injection as a secondary procedure was performed in eleven cases (79%). In the remaining three patients (21%), a symmetric facial appearance was achieved after the 1^st^ stage of treatment and judged the fat injection not needed. The mean pre-/postoperative FDI was 87.6 (SD 6.3), and 95.4 (SD 5.2) with a significant advance to facial symmetry (paired t-test, *p <* 0.001, Table 1). The intra-rater ICCs for the calculation of pre-/postoperative FDI were r = 0.96 and r = 0.94. The mean pre-/postoperative VAS was 7.2 (SD 1.7) and 3.8 (SD 2.4) respectively, with a significant improvement of the facial asymmetry (Mann-Whitney *U* test, *p* = 0.002, Fig 4). A high degree of internal consistency was observed for the facial asymmetry evaluation (Cronbach’s alpha value of 0.91).

**Table 1:** Patient demography.

Case	Age at surgery (years)	Right/ Left side	Male/ Female	Microtia	Previous surgery	Pruzansky- Kaban classification	Surgical procedures 1^st^ stage	Fat injection (ml) 2^nd^ stage	Preoperative FDI, %	Postoperative FDI, %
1	28.0	R	M	-	-	I	OGS MAR, MR, BFPR	Volume not recorded	96.0	102.4
2	19.6	R	M	+	-	I	OGS ASO	90	86.9	102.2
3	17.4	L	F	-	-	I	OGS ZR, MR, BFPR	53	84.1	99.6
4	24.0	R	F	-	-	I	OGS MAR	-	86.6	95.4
5	29.6	R	M	-	DOG	IIA	OGS	Volume not recorded	89.5	85.0
6	19.3	R	M	-	-	IIA	OGS BFPR	57	91.7	98.9
7	20.0	R	F	+	-	IIA	OGS	58	93.4	90.4
8	18.7	R	M	+	-	IIA	OGS	-	92.6	96.3
9	18.5	L	F	-	-	IIA	OGS ZR	47	83.4	96.9
10	19.9	L	F	-	-	IIA	OGS	Volume not recorded	85.5	96.1
11	19.6	L	F	+	-	IIA	OGS	18	90.0	97.0
12	20.6	L	F	+	-	IIB	OGS	-	95.7	99.8
13	21.2	L	F	+	DOG	IIB	OGS MAR, MR	25	77.3	88.3
14	27.8	R	M	-	-	IIB	OGS MAR	80	73.2	88.4
Average (SD)	21.7 (3.8)	R 8/ L 6	M 6/ F 8	-	-	I 4, IIA 7, IIB 3			87.6 (6.3) *	95.4 (5.2) *

**Abbreviations**: ASO, anterior segmental osteotomy; BFPR, buccal fat pad removal; DOG, distraction osteogenesis; FDI, facial surface area discrepancy index; MAR, mandibular angle resection; MR, masseter muscle reduction; OGS, orthognathic surgery including LeFort I, bilateral sagittal split osteotomy and genioplasty in all patients; ZR, zygoma reduction.

*: *p* < 0.001, paired t-test.

### Case example 1 (case 4)

**Fig 5:**
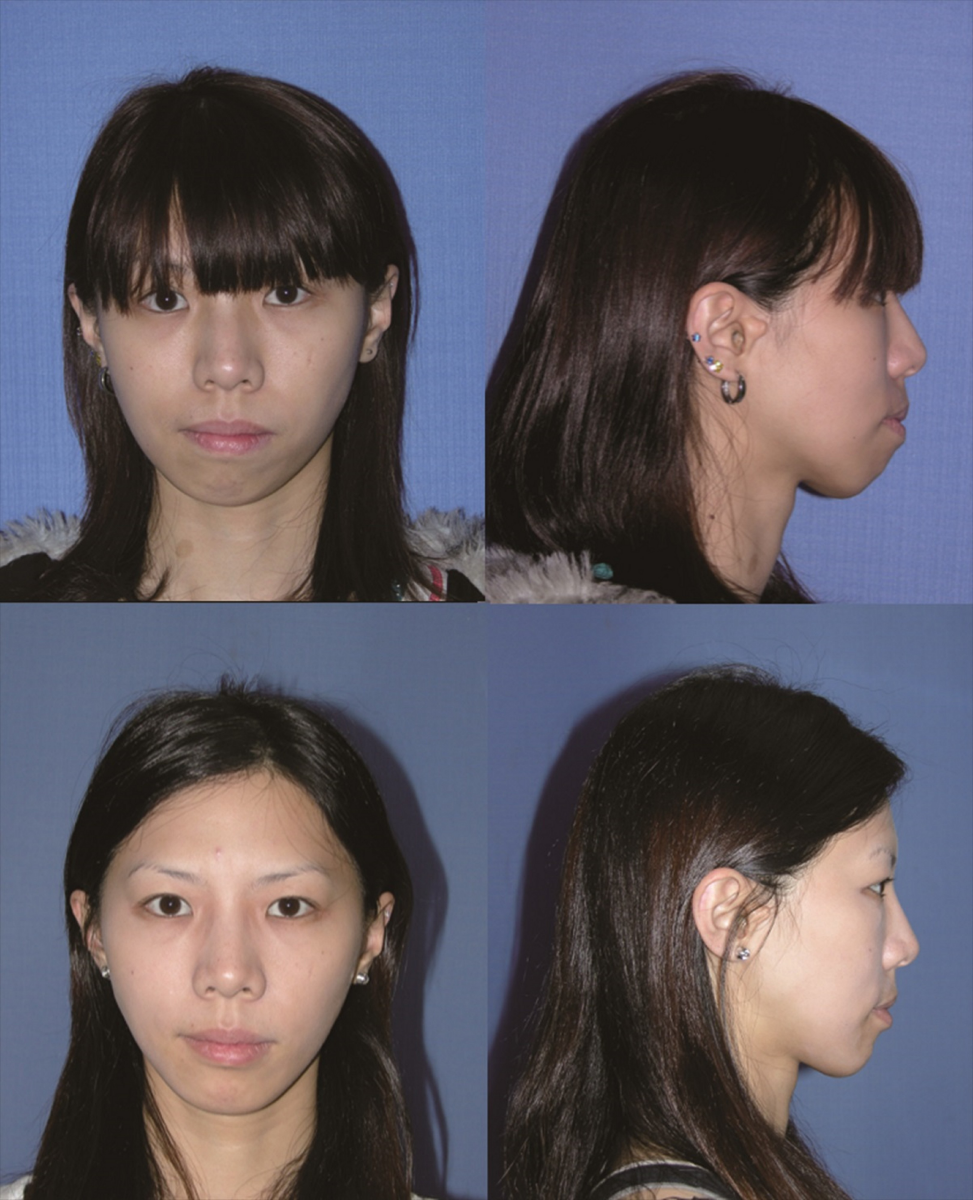
The 24-year-old female patient (case 4) was diagnosed with HFM (type I). Her natural head position was inclined to the right to compensate for her chin deviation. She was treated using Le Fort I, bilateral sagittal split osteotomy, genioplasty, and mandibular angle reduction, without a second stage of fat injection. Her natural head position adjusted spontaneously after surgery.

The 24-year-old female patient had HFM (type I) on the right side of her face (Fig 5). Her natural head position was inclined to the right side to compensate for her chin deviation. Regarding facial bone structures, the imaging study showed a small mandible, posterior cross bite, and canting of the occlusal plane. Around the mandibular angle area, there was a distinctive difference between the affected and non-affected sides. A combined approach with single-splint two-jaw OGS and facial bone contouring was performed, including Le Fort I, bilateral sagittal split osteotomy, genioplasty, and mandibular angle reduction. There was no postoperative complication. Her natural head position adjusted spontaneously after surgery. Soft tissue symmetry assessment was performed six months later, and the result turned out to be balanced in term of facial harmony. The patient was satisfied with the surgical improvement and did not need fat injection.

### Case example 2 (case 9)

**Fig 6:**
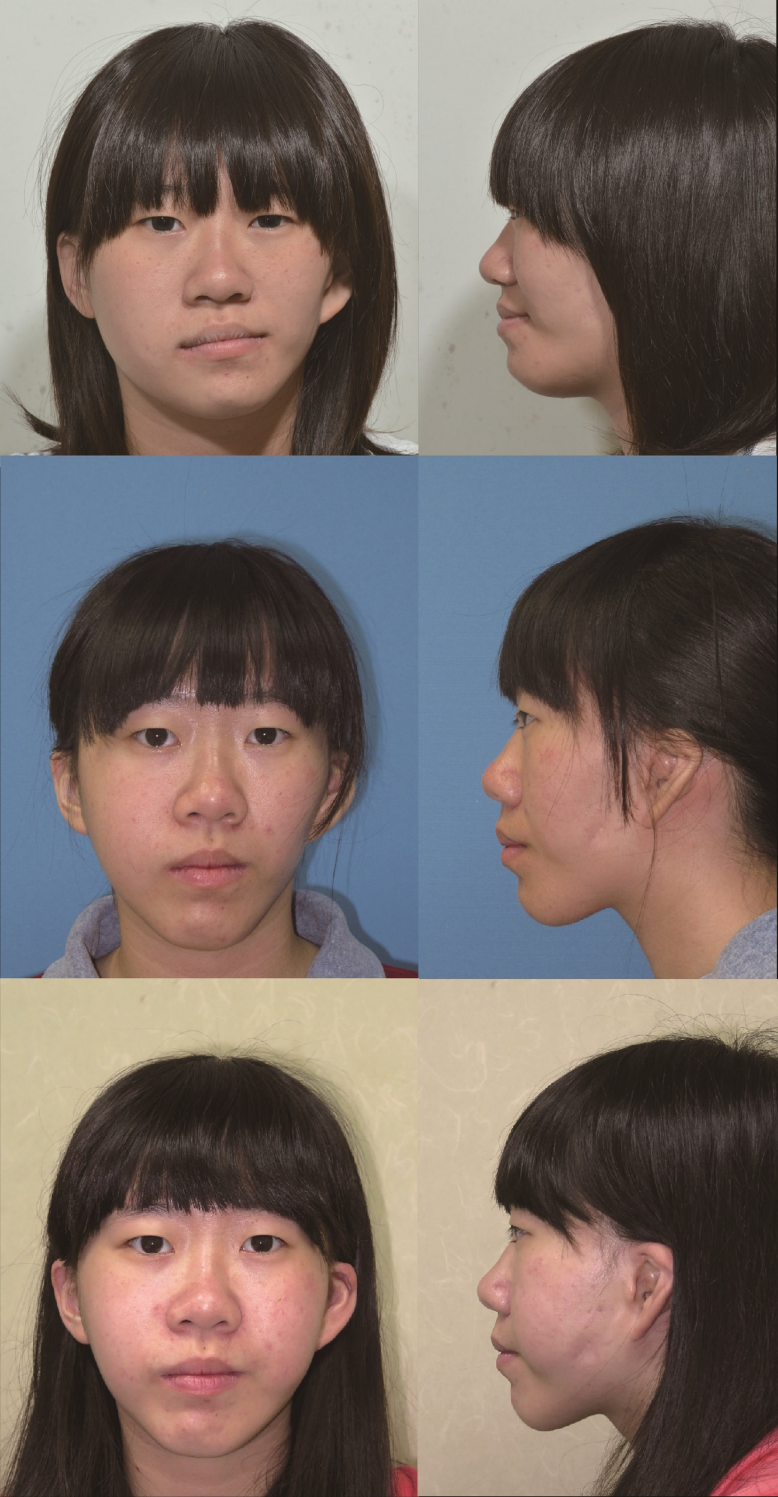
The patient (Case 9, type IIA) was an 18-year-old female who underwent OGS consisting of LeFort I, bilateral sagittal split osteotomy and genioplasty, combined with right zygoma reduction. 6 months postoperatively, soft-tissue augmentation for the deficient cheek using microautologous fat transplantation technique was performed with 47 ml of autologous fat. (Upper, before surgery; middle, after OGS; lower, after fat injection).

**Fig 7:**
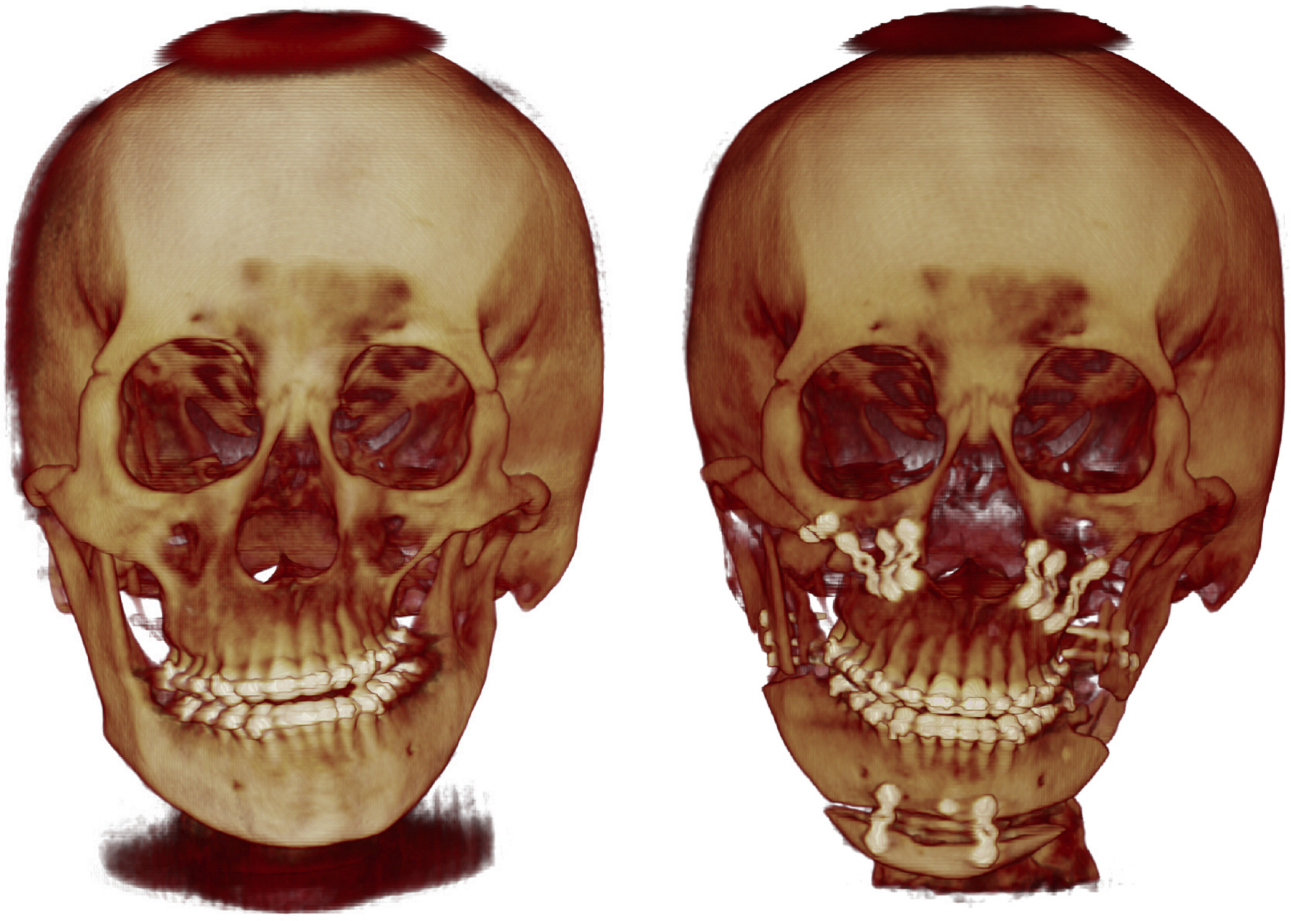
3-dimensional cone-beam computed tomogram of the patient case 9 in Fig 6. Preoperative image (left) showed facial bone deformity and asymmetry. The patient received two-jaw surgery, genioplasty, and right zygoma reduction (right).

The 18-year-old female patient was diagnosed with HFM (type IIA) affecting the left facial hemisphere from the middle to lower facial part (Fig 6). She presented with obvious deficiency of the left cheek soft tissue and notable lip canting. The CT showed moderate hypoplasia of the left mandible and a protruded zygoma on the non-affected side. The occlusal plane was canted upward to the left with posterior cross bite on both sides. She underwent two-jaw OGS consisting of LeFort I, bilateral sagittal split osteotomy and genioplasty, combined with facial contouring surgery of right zygoma reduction. Pre- and postoperative 3D cone-beam CT showed the deformity and correction of the facial bones (Fig 7). Six months after OGS, she underwent soft-tissue augmentation for the deficient cheek using the microautologous fat transplantation technique of fat injection. She recovered well from both surgeries with a substantial improvement of facial appearance, and was satisfied with the result.

### Case example 3 (Case 13)

**Fig 8:**
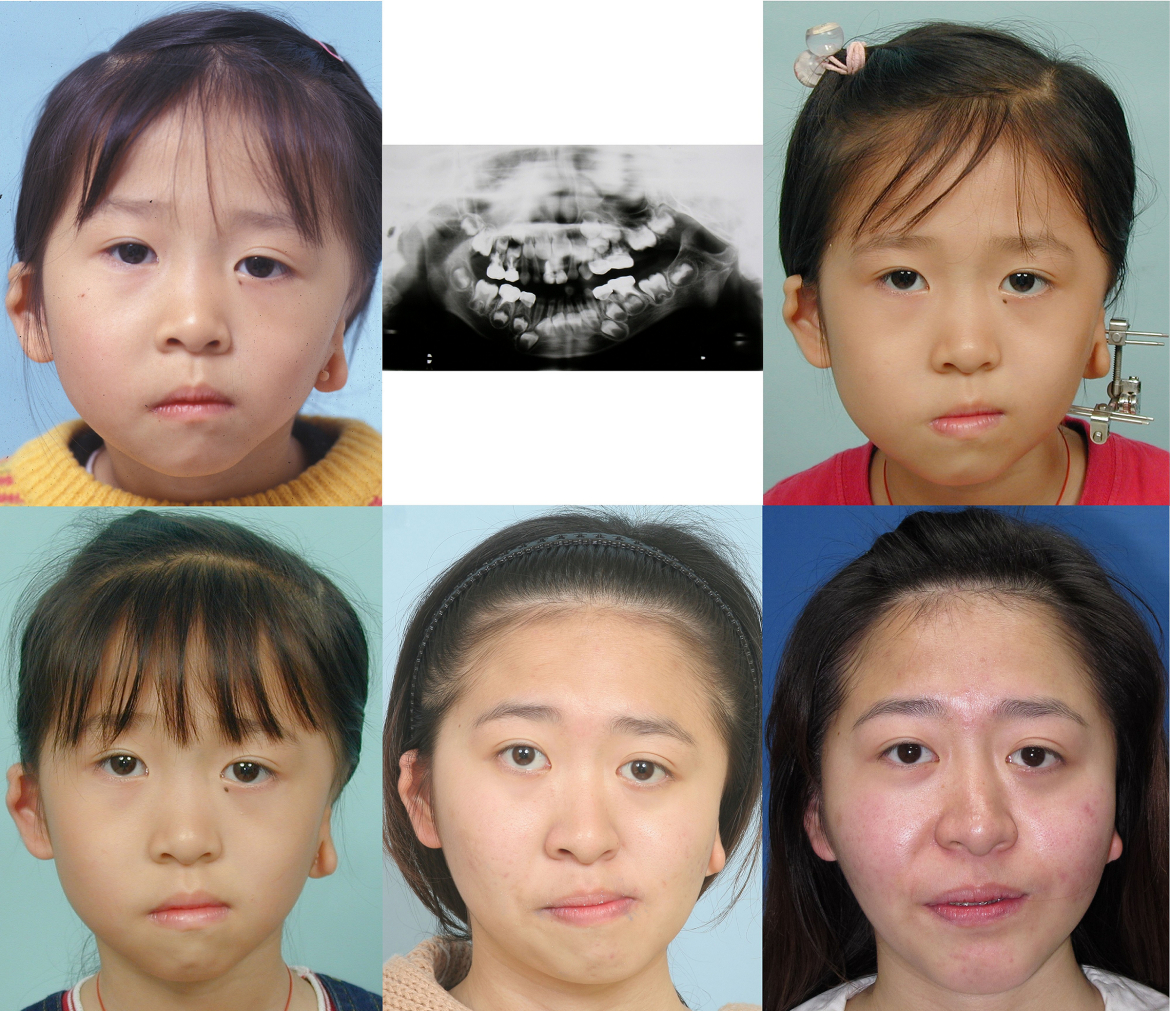
A female patient with Type IIB hemifacial microsomia involving the left side of face (case 13). She was followed up at 5 years of age (above left and middle). The panorex X-ray showed hypoplastic and inferiorly displaced condyle without adequate glenoid fossa. She was 7 years 9 months of age during the distraction osteogenesis (above right). Facial appearance was improved at 8 years 6 months of age (below left). Significant facial asymmetry was noted at 21 years 1 month of age (below middle). She received two stages of surgical correction. The facial appearance was improved at 22 years 2 months of age (below right).

**Fig 9:**
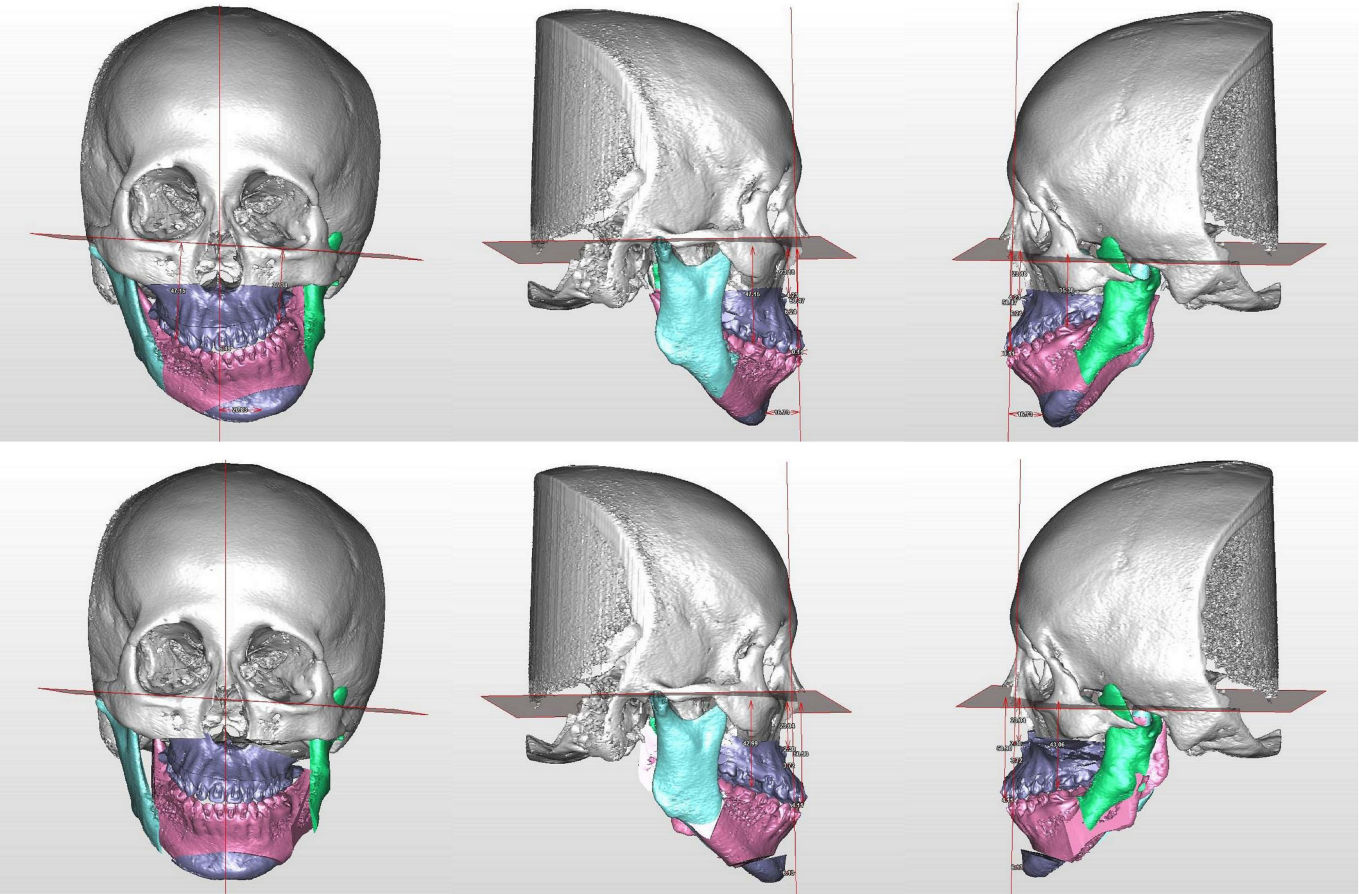
Three-dimensional surgical simulation for the case 13. Preoperative views were shown in the upper row. There was prominent asymmetry in the maxilla and mandible with occlusal canting. Part of the left zygomatic arch was missing. The ramus was short, but was thick after the previous distraction osteogenesis. The left condyle was hypoplastic and displaced inferiorly, medially and anteriorly. There was no proper glenoid fossa. The articulation was judged acceptable for orthognathic surgery. The surgical simulation and planning was shown in the lower row.

This patient had type IIB hemifacial microsomia (Fig 8). Her left side mandible and soft tissue were hypoplastic. She received distraction osteogenesis when she was 7 years old. At 21 years of age, significant facial asymmetry was noted (Fig 8, lower middle). Three-dimensional simulation was performed (Fig 9), and she underwent orthognathic surgery with LeFort I, bilateral sagittal split osteotomy, genioplasty, as well as mandibular angle and masseter muscle reduction on the right side. Fat injection was performed 9 months later as the second stage. She was happy with the results (Fig 8, lower right).

## Discussion

Application of bone surgery including LeFort I osteotomy and bilateral sagittal split osteotomy was previously reported for HFM patients with the goal of aesthetic and functional improvement [20, 21]. However, no treatment consensus has been established regarding the optimal timing for the surgery, type of surgery, and whether distraction osteogenesis should be used [10, 22–24]. The treatment protocols are varied. Our concept for HFM management is to perform surgery on skeletally mature patients with Pruzansky-Kaban type I and II deformity [24]. Aggressive management in the growing age may not guarantee long-term symmetry and aesthetic outcome. Two of our cases received skeletal distraction at younger age, but facial asymmetry persisted requiring OGS. Definitive surgery after the growth spurt ensures stable and persistent occlusal and facial outcome [24]. However, we excluded patients with Pruzansky-Kaban type III deformity for this protocol because these are severe cases who require more interventions such as rib graft, distraction osteogenesis or free flap surgery. It is to be noted that patients with type IIB could have rudimentary condyle with inadequate or no articulation between the ramus and temporal bone. In such situation, reconstruction of the temporomandibular joint sould be performed before consideration of orthognathic surgery [13].

The anatomical distortion should be addressed with CT imaging before surgery. It is well known that the size of the mandible, the shape and location of the temporomandibular joint, the size of the masseter muscle and the parotid gland are affected to various extents in HFM patients. Other anatomical variations include dysmorphogenesis of the temporal bone which can affect the styloid foramina of the facial nerve [25], hypoplasia or aplasia of muscles [26, 27], and variable intra-bony courses and exits of the inferior alveolar nerve [28]. Facial scoliosis and hypoplasia can be seen on the affected side of the face. The surgical goal of OGS is repositioning the distorted structures to achieve a balanced and symmetric appearance, and it is therefore important to consider the ideal yaw and roll rotation to balance the deficient side [29]. On the other hand, excessive rotation can potentially produce premature contacts in various areas which hinder stable fixation. The introduction of 3D computer simulation enables visualizing and addressing these problems before surgery, including posterior collision of the maxillary segments and premature contacts between the mandibular segments [15]. Moreover, adjusting premature contacts in accordance with yaw and roll movements can be utilized to augment the width of the ramus area for balancing the jaw line and cheek contour by keeping the space between proximal and distal segments (Fig 1).

In terms of soft-tissue, the correction of the occlusal plane and bone configuration does not promise to achieve sufficient facial symmetry. Especially for patients with prominent soft tissue discrepancy, OGS can accentuate the deficiency around the jawline on the affected side due to shifting of the maxilla and mandible and stretching out of the soft tissue regardless of bony augmentation (Fig 1). Our integrated approach reduces this discrepancy, and the subsequent soft tissue augmentation becomes more convenient. For some patients, the corner of the mouth tends to stay higher on the affected side even after the correction of the facial bones. We have noticed that soft tissue augmentation, fat injection in this study, can improve or correct the lip cant. This observation also happen in other patients with facial asymmetry. Although various surgical techniques such as dermal-fat grafting [30], local or free flap augmentation [31,32], and artificial materials such as silicone, hydroxyapatite and Medpor can provide facial volume augmentation [33–35], we prefer to use fat injection with the microautologous fat transplantation (MAFT) gun (Dermato Plastica Beauty Co., Ltd, Kaohsiung, Taiwan) because of reduced invasiveness, morbidity, and ease of repeatability [11]. Satisfactory results were obtained from this appraoch with less surgical morbidity.

Limitations to the present report exist. The study design was retrospective and is therefore subject to confounding errors in terms of methodology. Our assessment tool for asymmetry was a 2D measurement of the facial area on a standardized clinical photo, which has systematic errors in nature compared to 3D volumetric imaging. 3D photogrammetry has been validated for the assessment of cranio-maxillofacial measurements [36]. Only two cases in this series had 3D photographic imaging for fat injection. Although it has shown potential to be the most objective method of assessment, a 2D photo evaluation is still easily carried out and sensitive to detect discrepancy from both sides. Also, patients tend to be concerned about their appearance in the mirror or frontal photos on social platforms. Accordingly, concerns of facial appearance should be addressed from a frontal view. In our dataset, we found significant differences between the pre- and postoperative appearance regarding the FDI and perceived symmetry, indicating the significant improvement. Considering potential methodological errors, the ideal study design should be a prospective study using a 3D volumetric technique to assess the effectiveness of the treatment protocol. However, the comprehensive approach using OGS and subsequent fat injection instead of a single surgical procedure for HFM is reasonable and beneficial considering the complexity of the disease. It is observed that these groups of patients are best treated after growth is completed and that patients treated with distraction quite frequently required additional OGS and facial recontouring procedures to achieve an optimal result. Although the literature is divided on this, most of the evidence appears to indicate that the deformity in HFM does not worsen significantly during growth and unless there is a functional problem we believe the best time and way to treat is as described in this study [37–39]. This step-wise treatment protocol can enhance the patient’s quality of life as an effective approach in our subset of HFM patients, which can be assessed using patient-reported outcome instruments as a further study in the future.

## Supporting information

S1 File VAS for hemifacial microsomia patients. (PPTX)
