# Supplementary material for: An integrated surgical protocol for adult patients with hemifacial microsomia: Methods and outcome
Source: PLoS One. 2017 Aug 4;12(8):e0177223. doi: 10.1371/journal.pone.0177223 (PMC5544248; doi:10.1371/journal.pone.0177223)
Supplement: S1 File — (PPTX) [file pone.0177223.s001.pptx]

## Slide 1
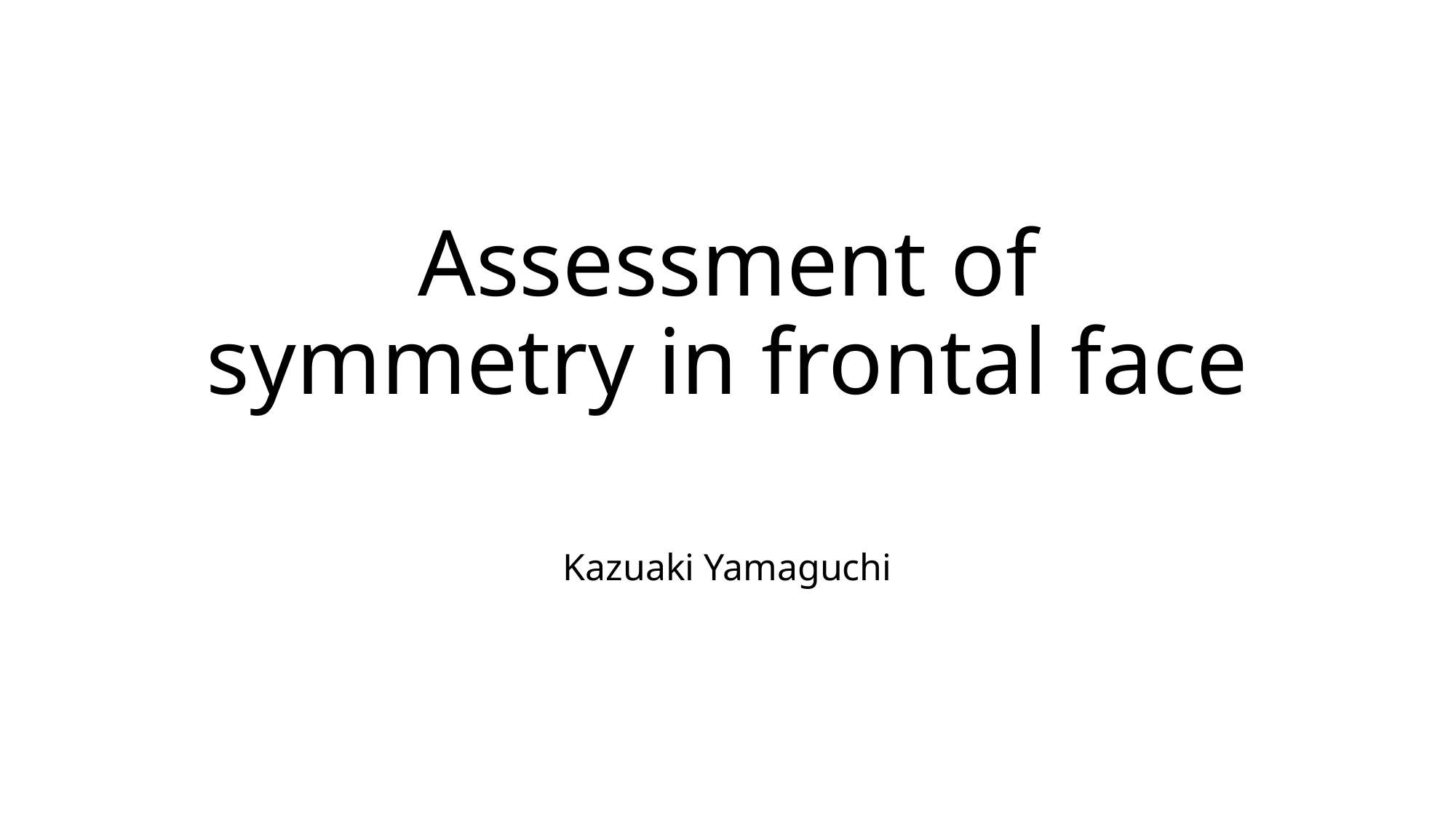

# Assessment of symmetry in frontal face
Kazuaki Yamaguchi

## Slide 2
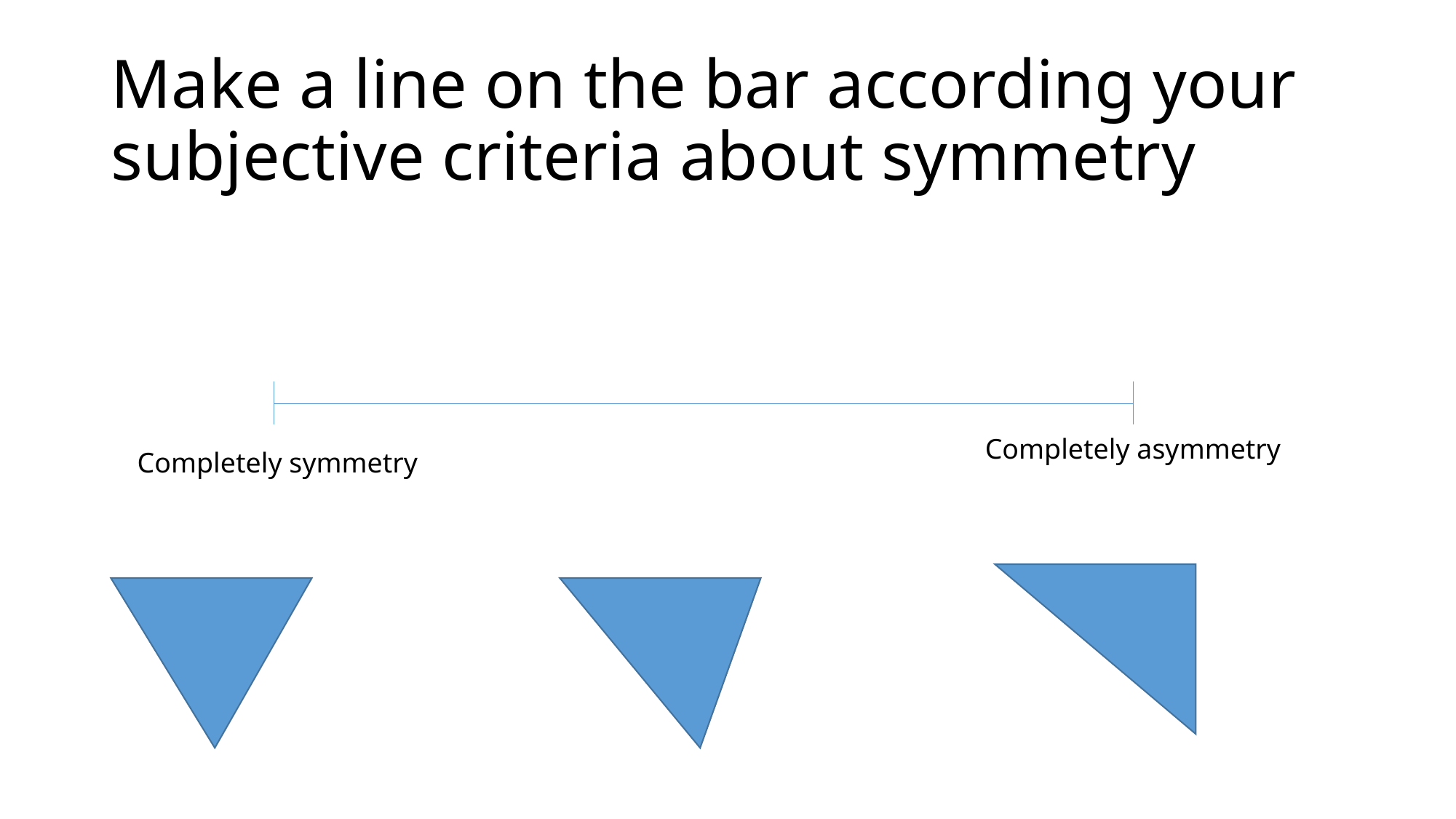

# Make a line on the bar according your subjective criteria about symmetry
Completely asymmetry
Completely symmetry

## Slide 3
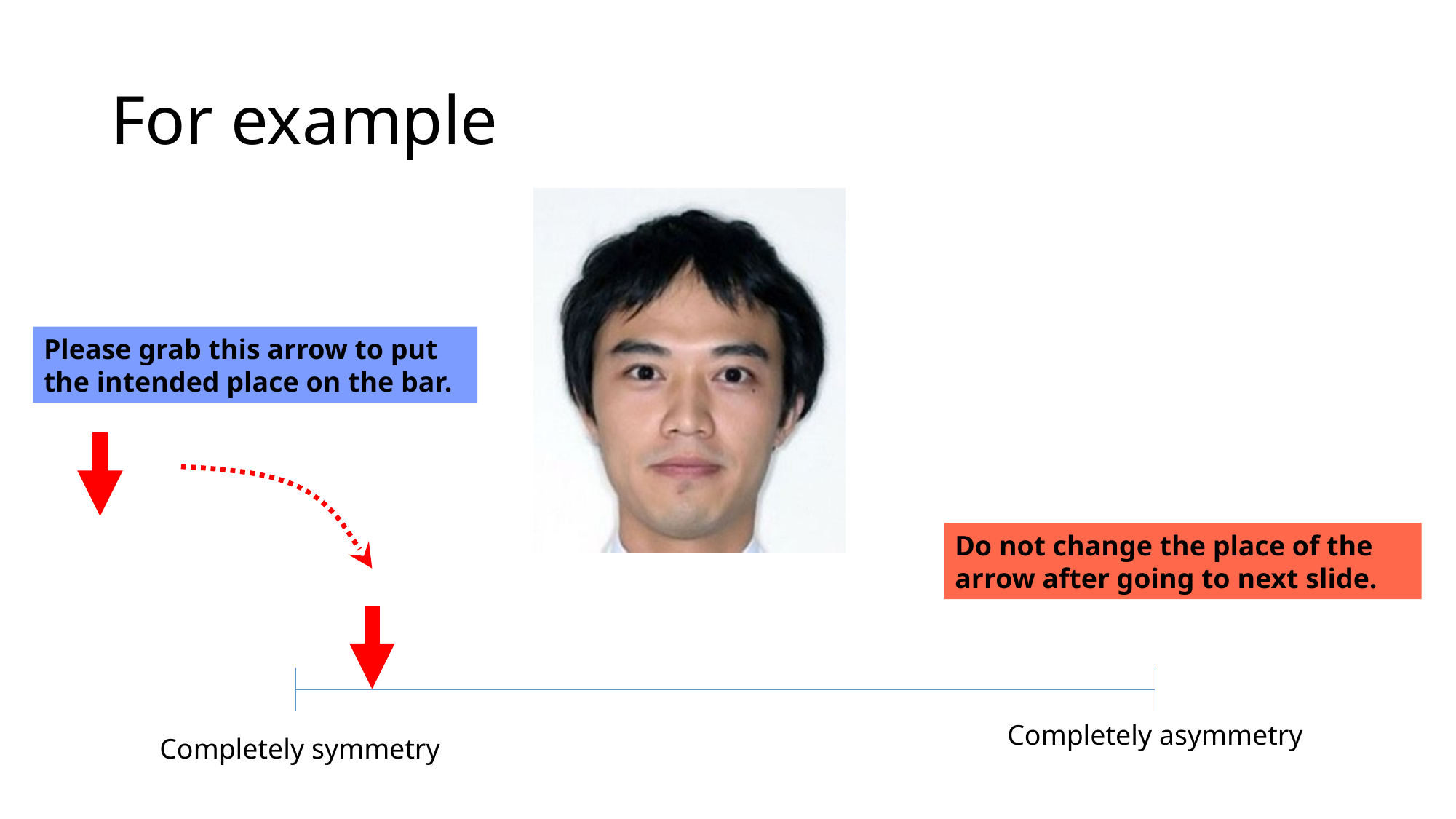

# For example
Please grab this arrow to put the intended place on the bar.
Do not change the place of the arrow after going to next slide.
Completely asymmetry
Completely symmetry

## Slide 4
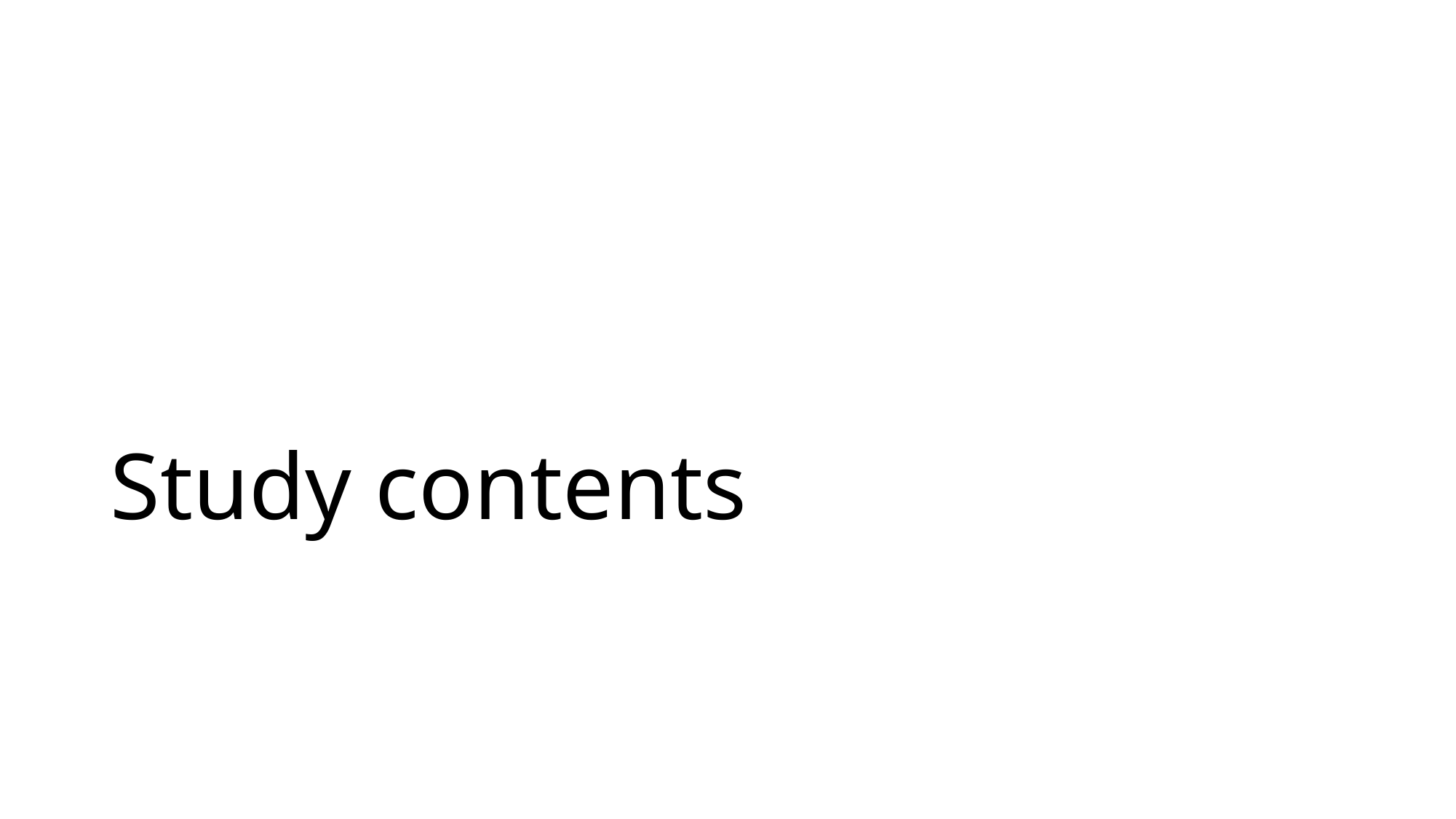

# Study contents

## Slide 5
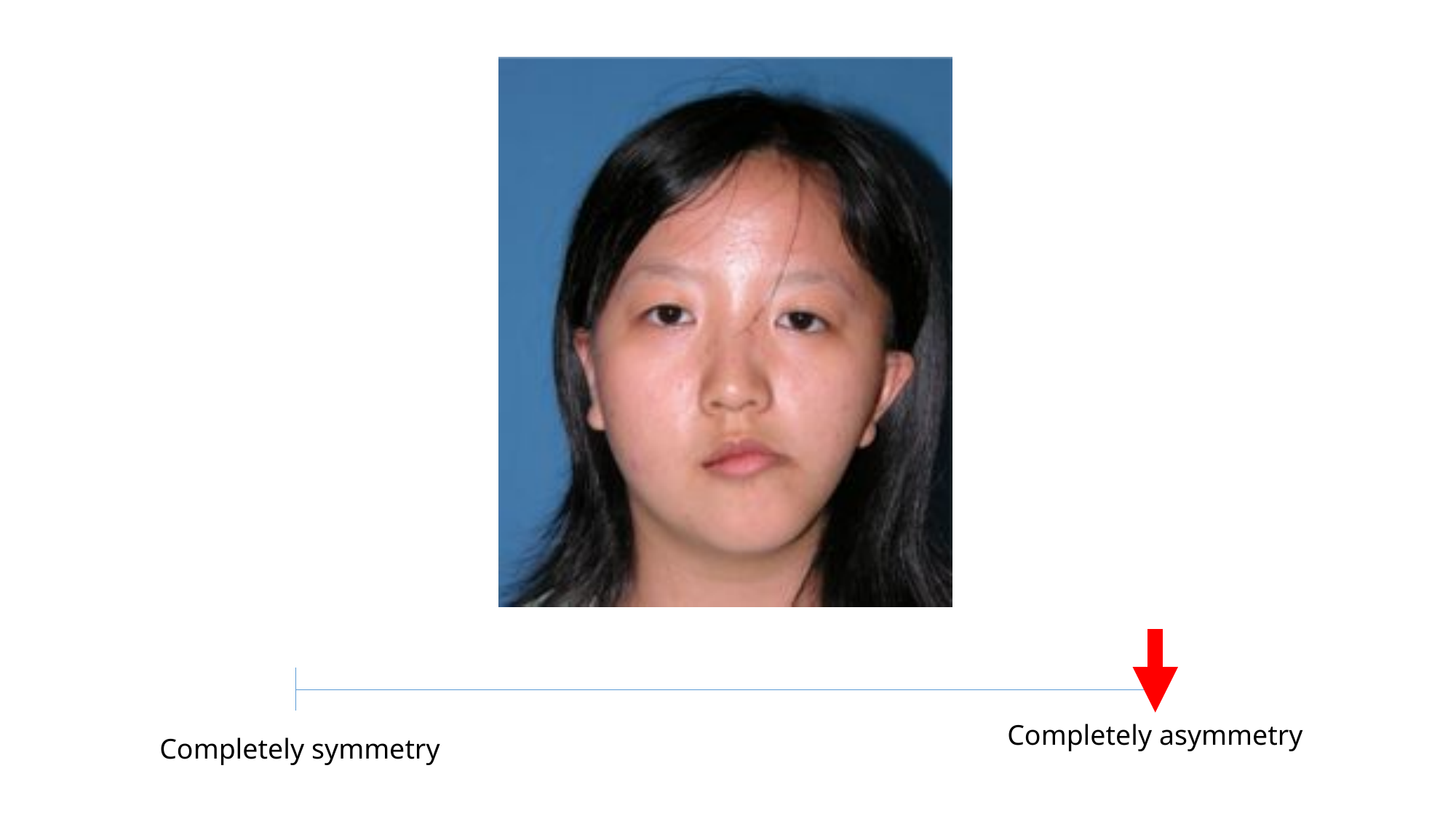

Completely asymmetry
Completely symmetry

## Slide 6
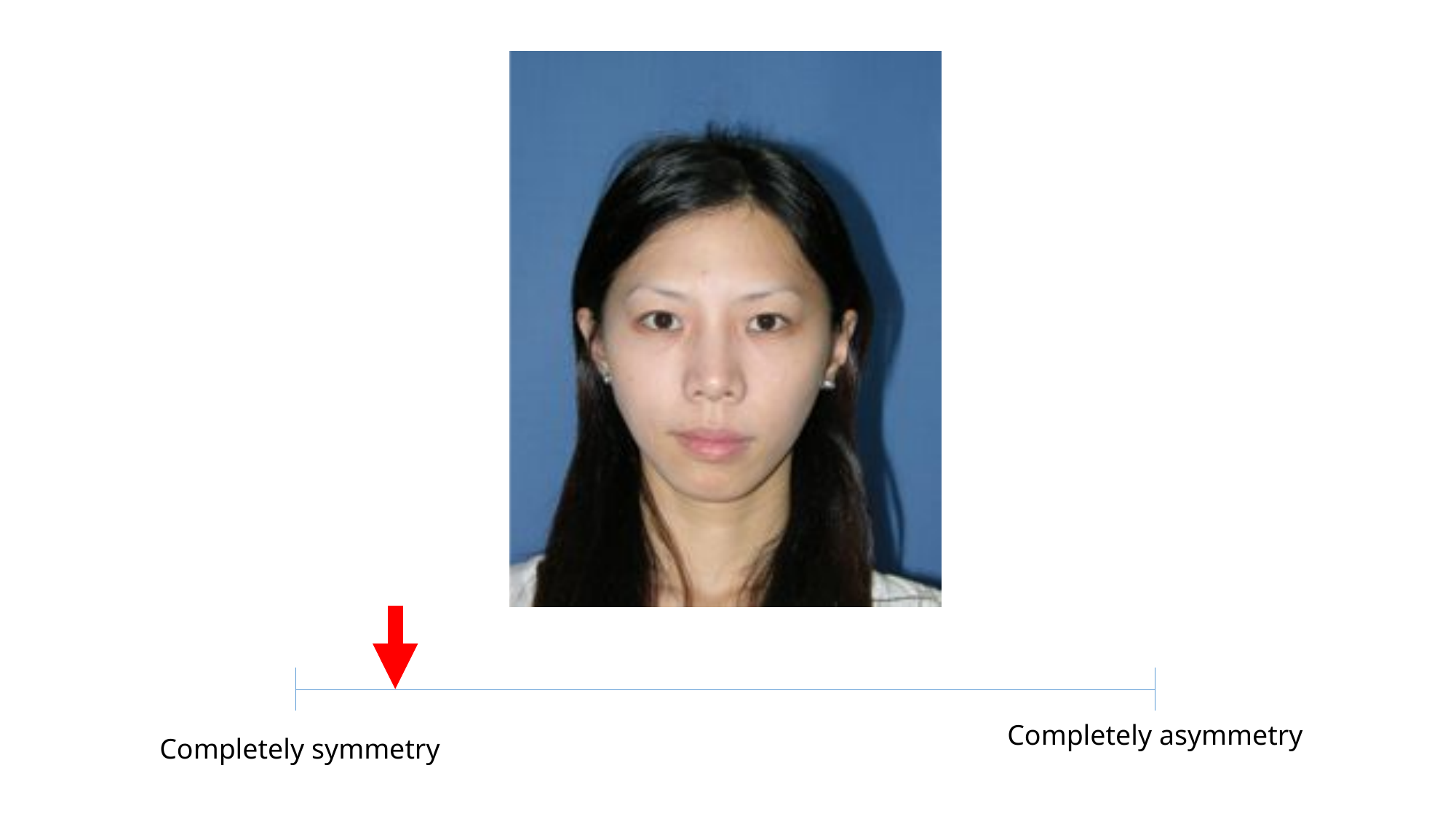

Completely asymmetry
Completely symmetry

## Slide 7
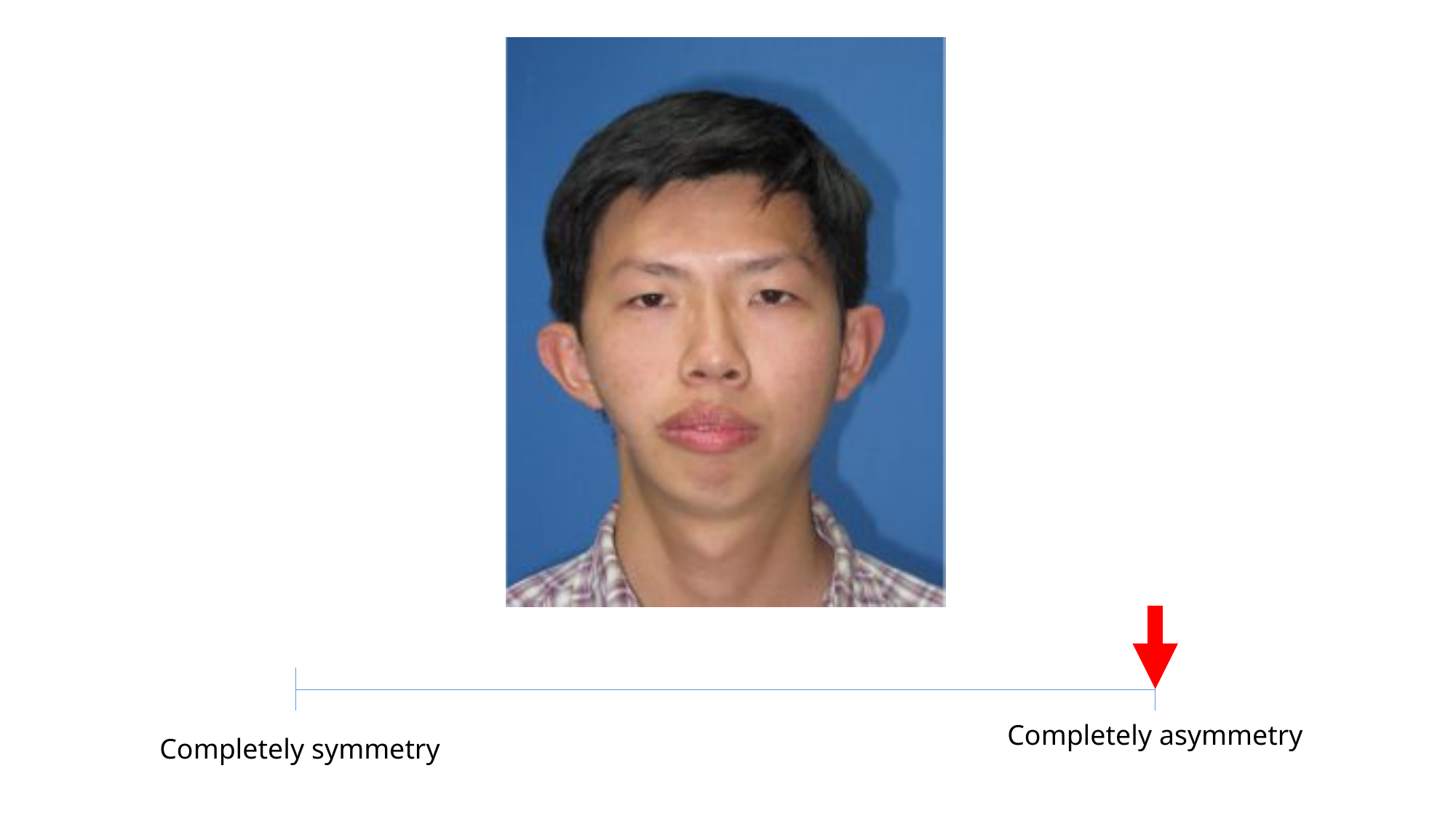

Completely asymmetry
Completely symmetry

## Slide 8
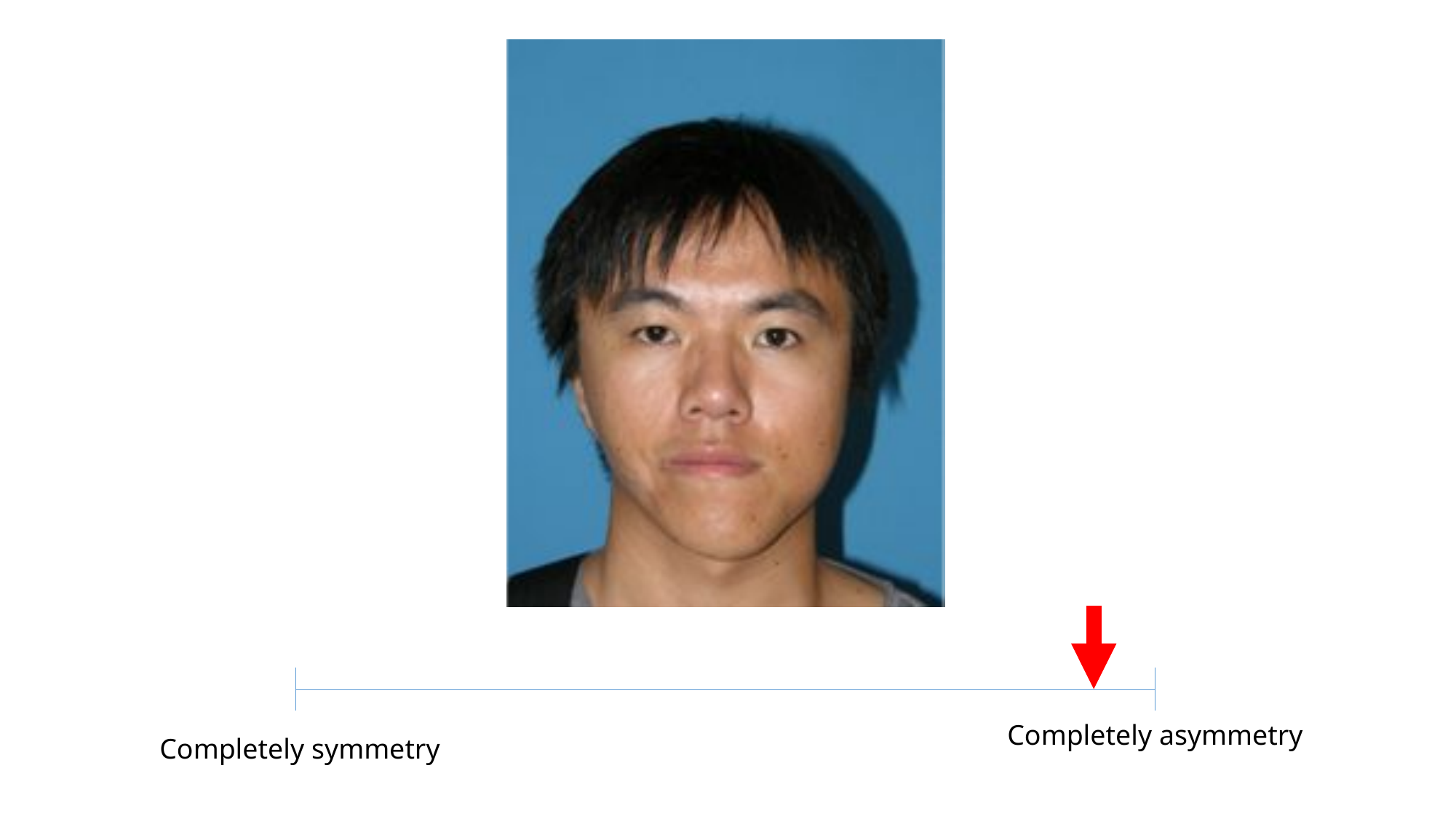

Completely asymmetry
Completely symmetry

## Slide 9
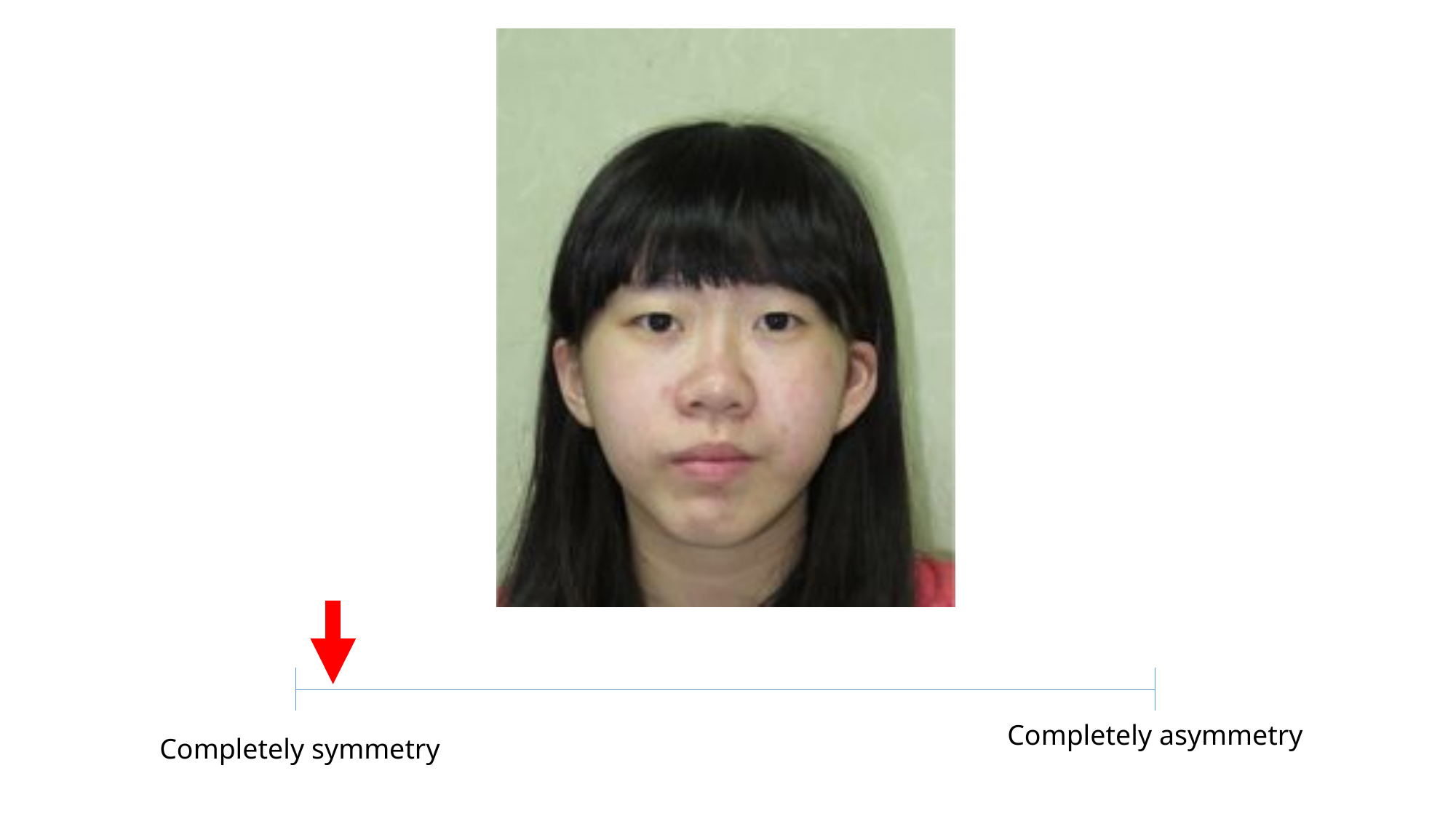

Completely asymmetry
Completely symmetry

## Slide 10
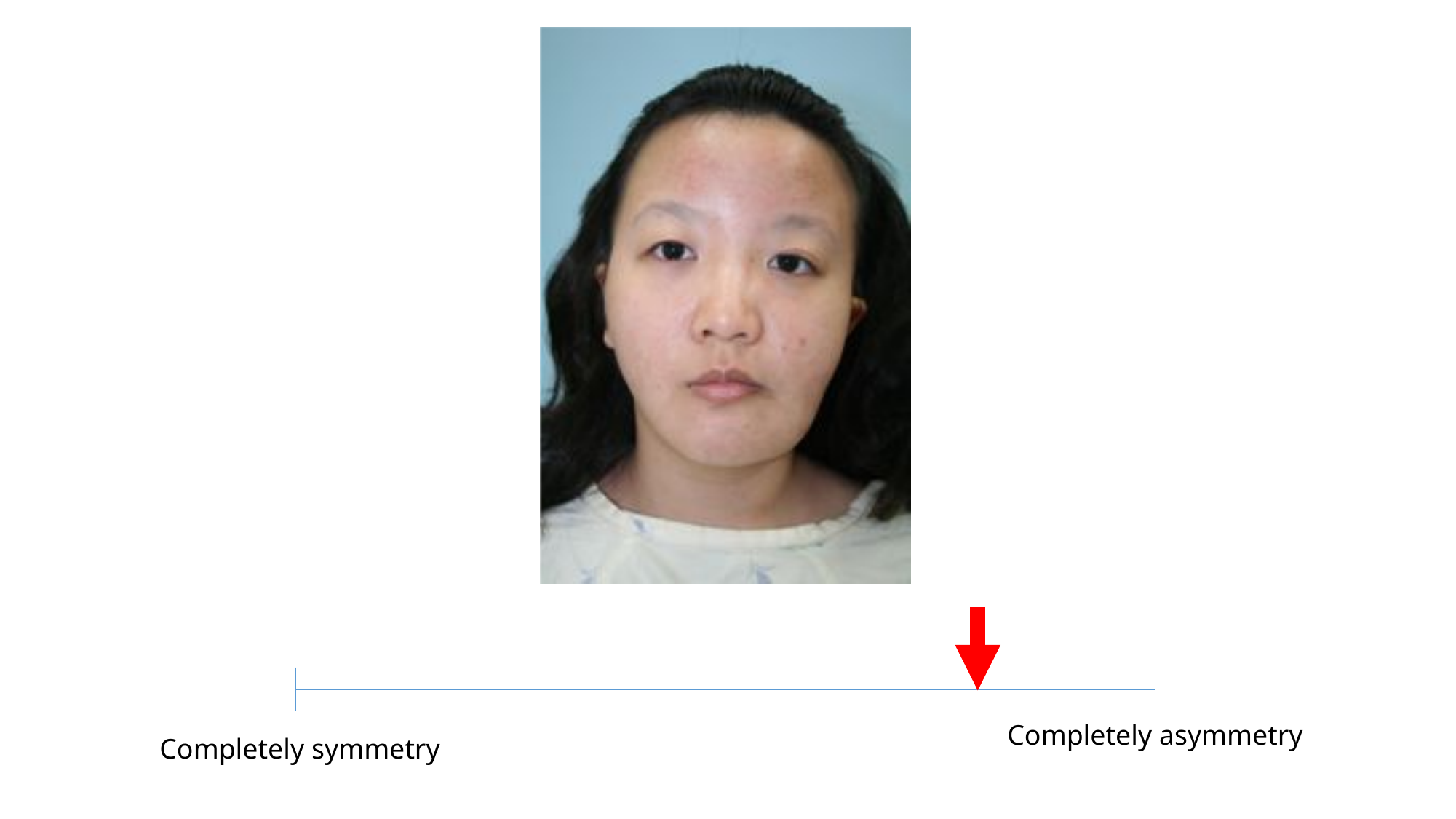

Completely asymmetry
Completely symmetry

## Slide 11
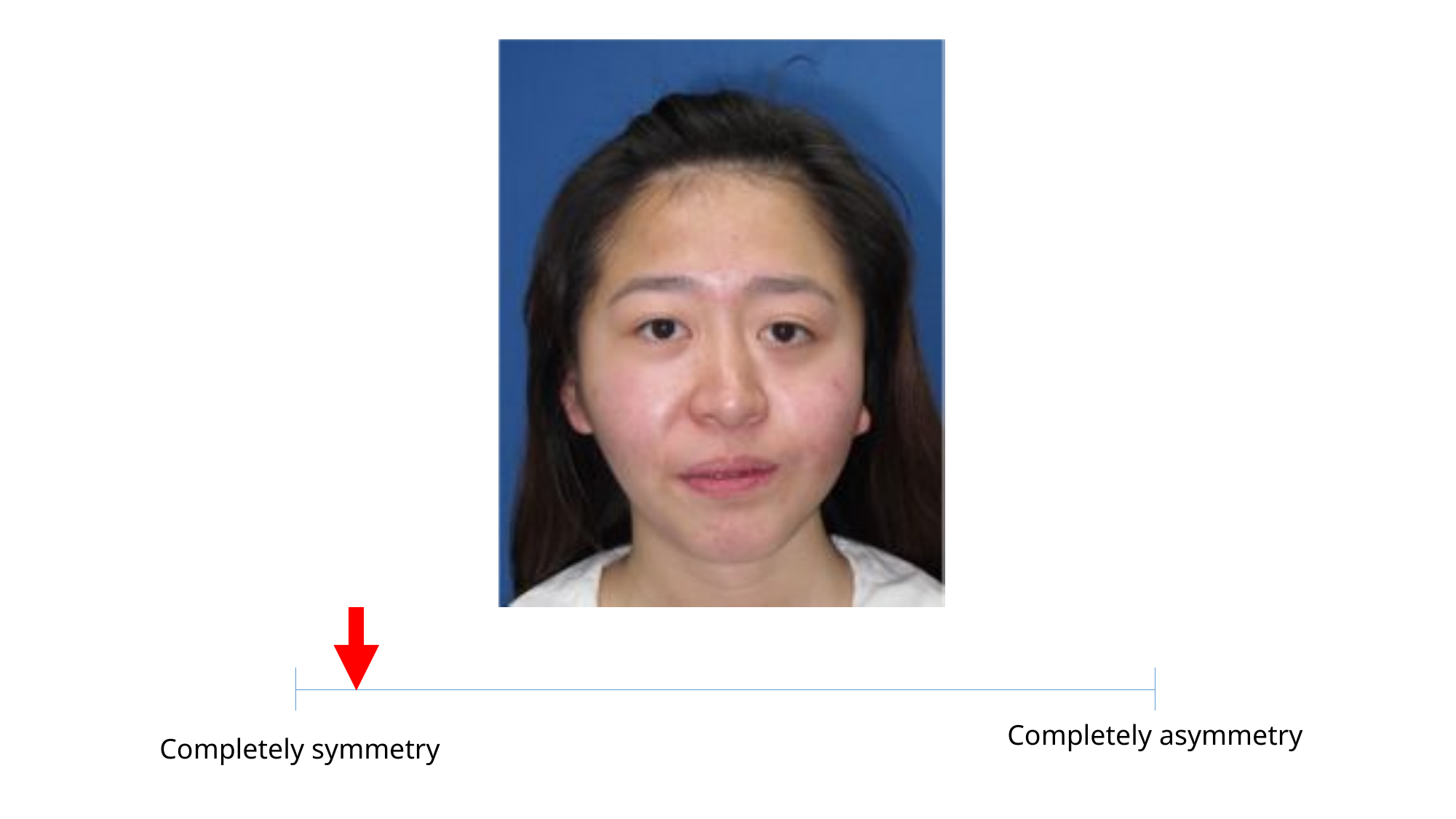

Completely asymmetry
Completely symmetry

## Slide 12
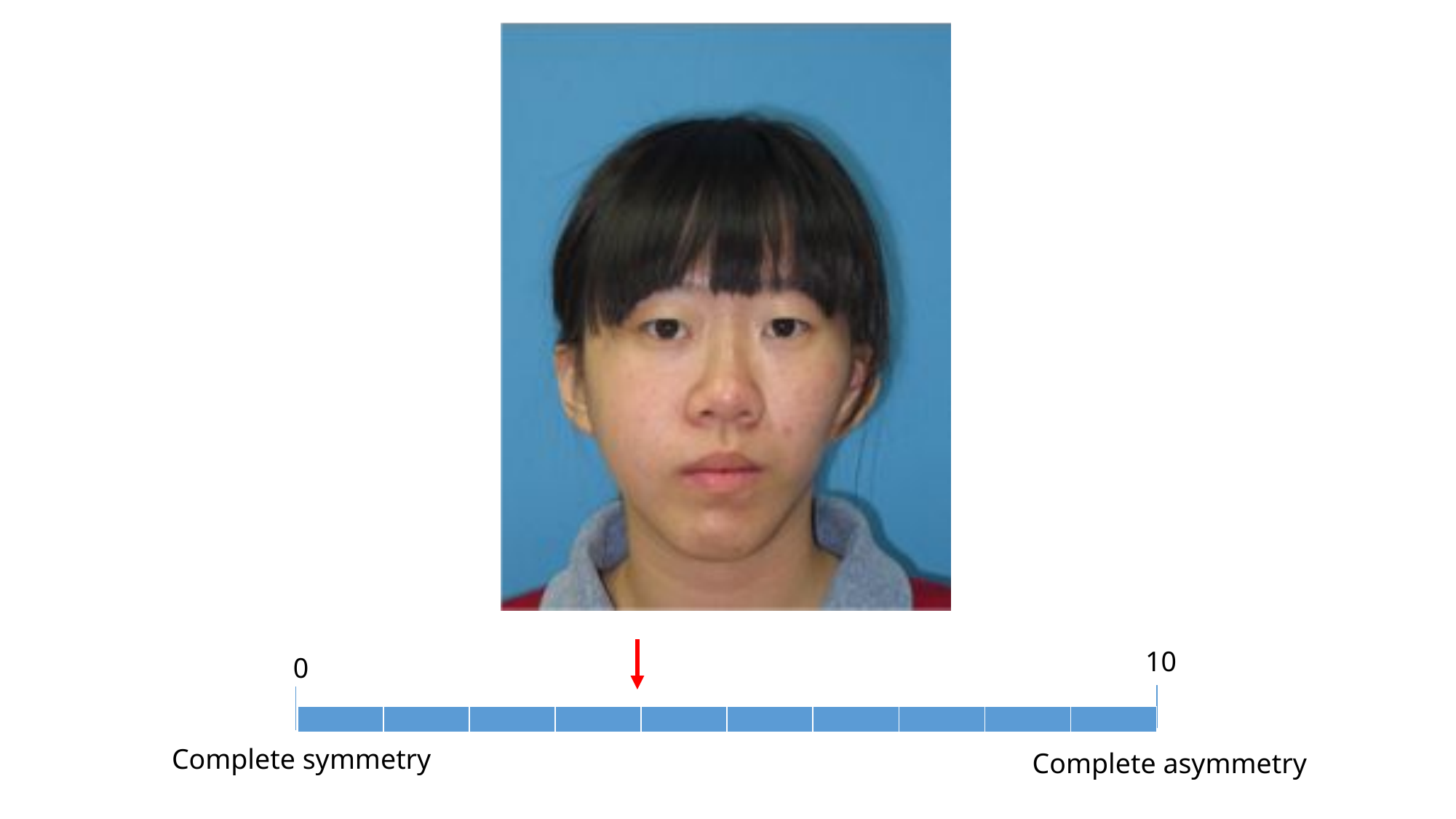

10
0
Complete symmetry
Complete asymmetry
| | | | | | | | | | |
| --- | --- | --- | --- | --- | --- | --- | --- | --- | --- |

## Slide 13
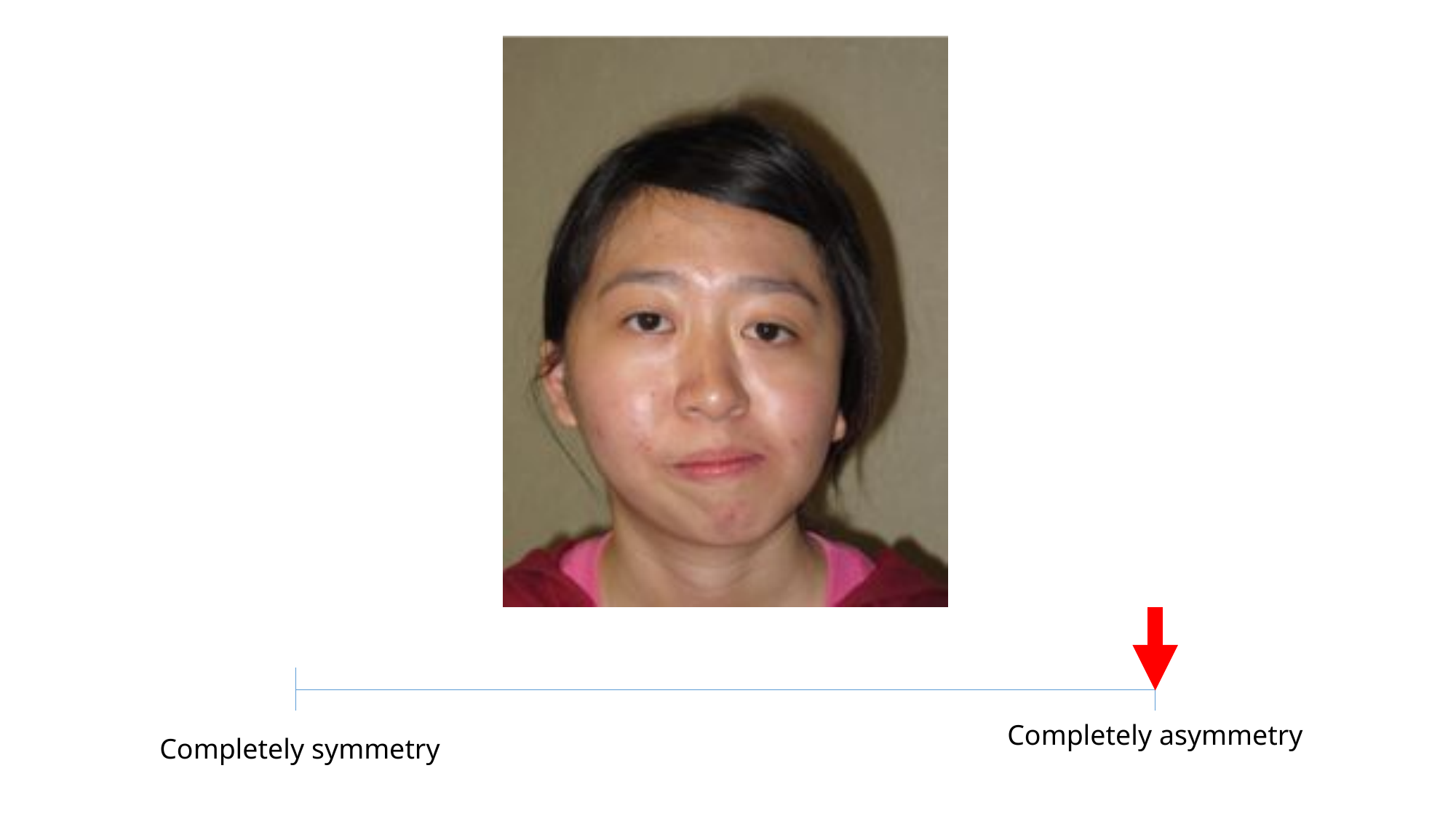

Completely asymmetry
Completely symmetry

## Slide 14
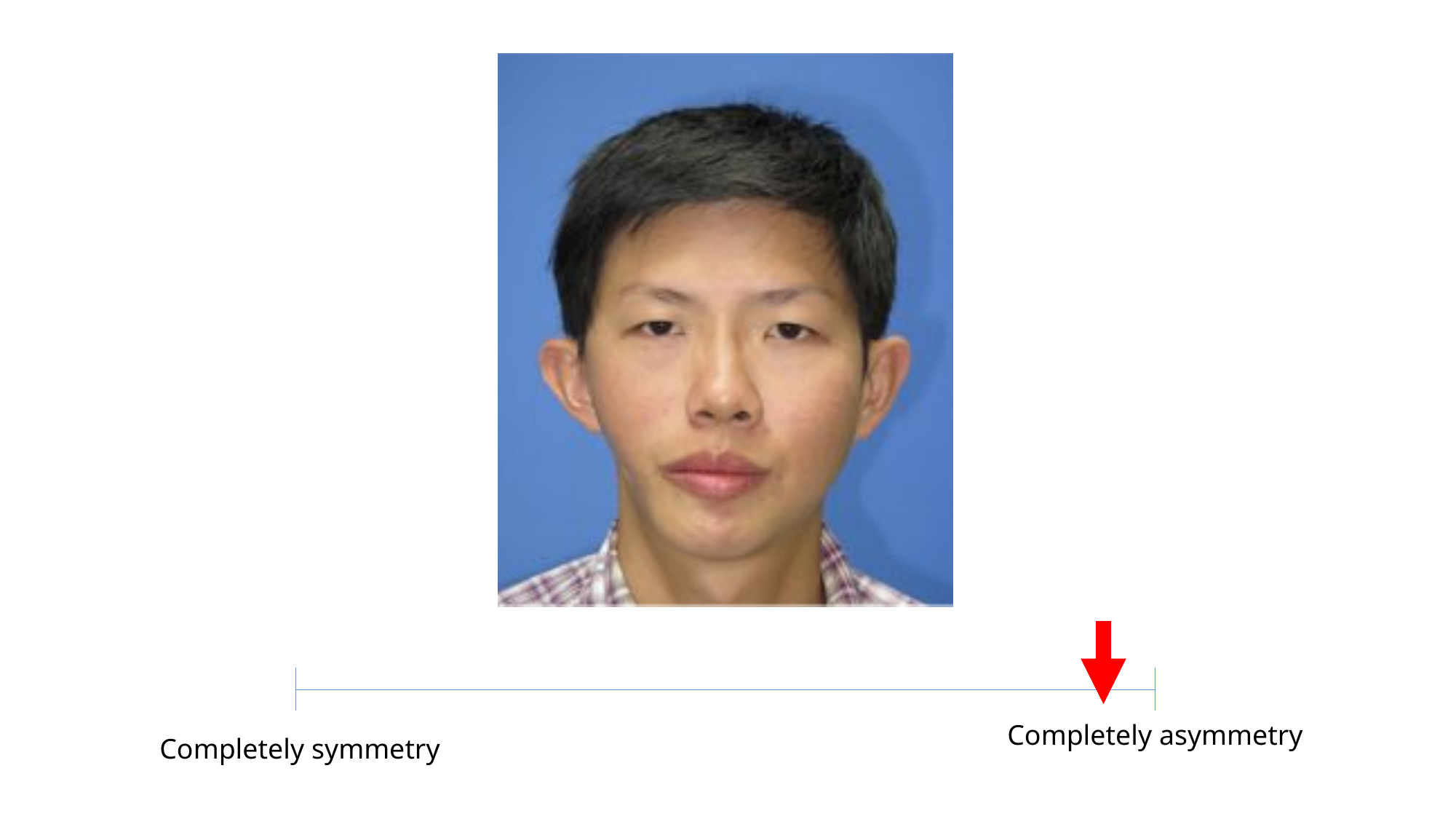

Completely asymmetry
Completely symmetry

## Slide 15
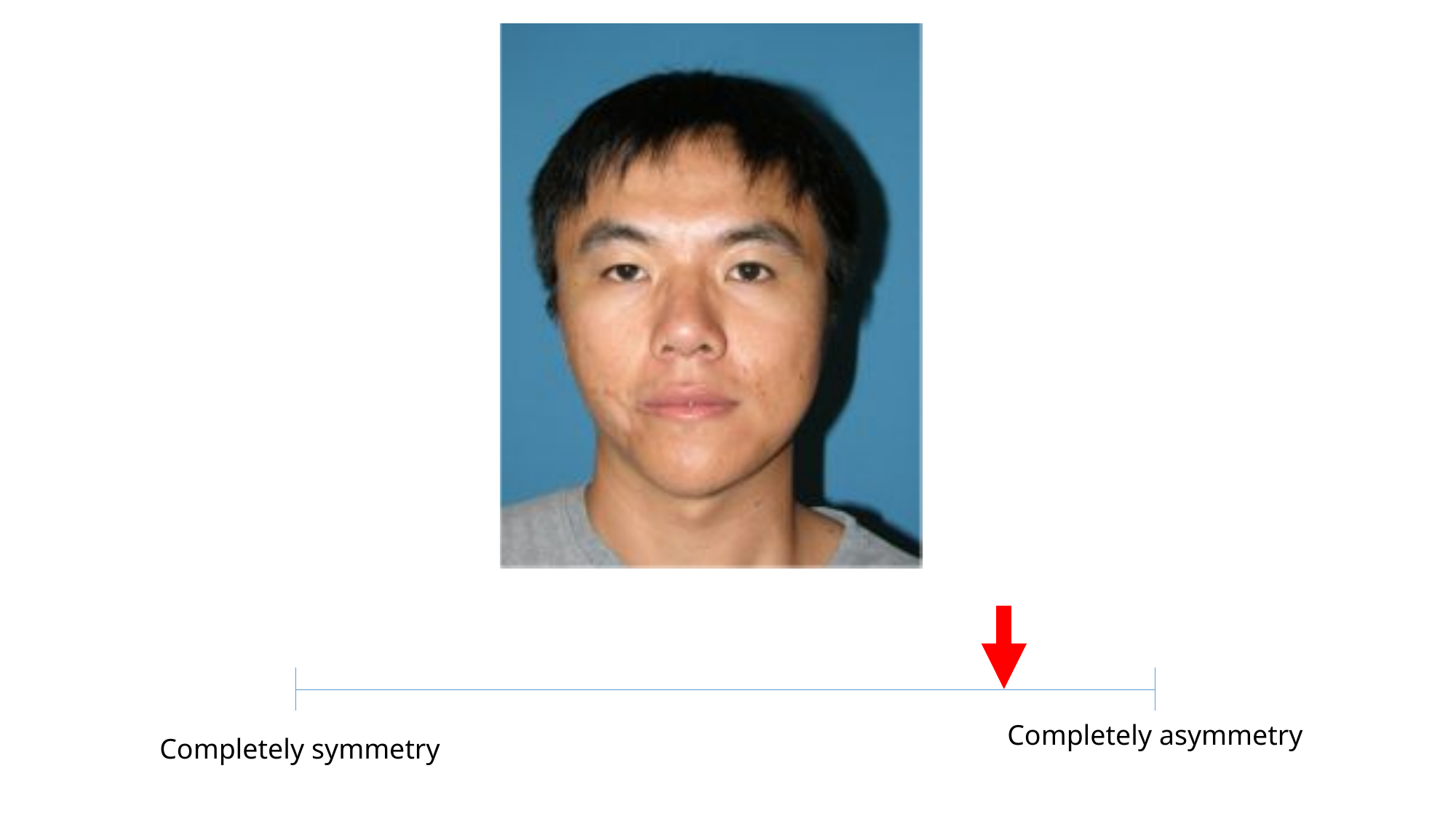

Completely asymmetry
Completely symmetry

## Slide 16
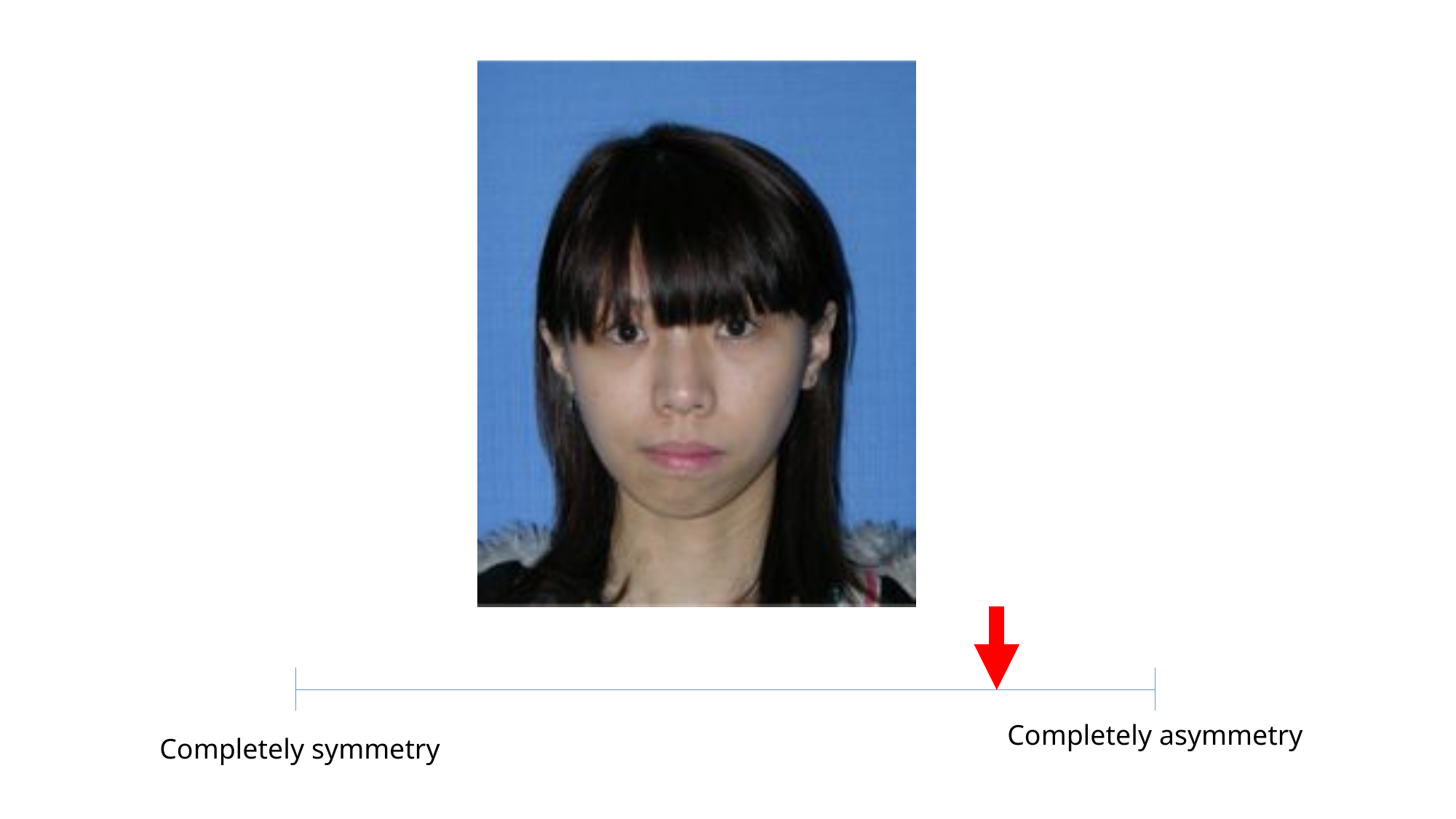

Completely asymmetry
Completely symmetry
